# Seven-year follow-up of durability and safety of AAV CNS gene therapy for a lysosomal storage disorder in a large animal

**DOI:** 10.1016/j.omtm.2021.09.017

**Published:** 2021-10-05

**Authors:** Sara Marcó, Virginia Haurigot, Maria Luisa Jaén, Albert Ribera, Víctor Sánchez, Maria Molas, Miguel Garcia, Xavier León, Carles Roca, Xavier Sánchez, Joan Bertolin, Jennifer Pérez, Gemma Elias, Marc Navarro, Ana Carretero, Martí Pumarola, Anna Andaluz, Yvonne Espada, Sonia Añor, Fatima Bosch

**Affiliations:** 1 Center of Animal Biotechnology and Gene Therapy, Edifici H, Universitat Autònoma de Barcelona, 08193 Bellaterra, Spain; 2 Department of Biochemistry and Molecular Biology, Universitat Autònoma de Barcelona, 08193 Bellaterra, Spain; 3 Department of Animal Medicine and Surgery, Universitat Autònoma de Barcelona, 08193 Bellaterra, Spain; 4 Hospital Clínic Veterinari, Universitat Autònoma de Barcelona, 08193 Bellaterra, Spain; 5 CIBER de Diabetes y Enfermedades Metabólicas Asociadas (CIBERDEM), 28029 Madrid, Spain

**Keywords:** adeno-associated viral vector, central nervous system, gene therapy, lysosomal storage disease, mucopolysaccharidosis type IIIA, durability, safety, cerebrospinal fluid, brain, dorsal root ganglia

## Abstract

Delivery of adeno-associated viral vectors (AAVs) to cerebrospinal fluid (CSF) has emerged as a promising approach to achieve widespread transduction of the central nervous system (CNS) and peripheral nervous system (PNS), with direct applicability to the treatment of a wide range of neurological diseases, particularly lysosomal storage diseases. Although studies in small animal models have provided proof of concept and experiments in large animals demonstrated feasibility in bigger brains, there is not much information on long-term safety or durability of the effect. Here, we report a 7-year study in healthy beagle dogs after intra-CSF delivery of a single, clinically relevant dose (2 × 10^13^ vg/dog) of AAV9 vectors carrying the canine sulfamidase, the enzyme deficient in mucopolysaccharidosis type IIIA. Periodic monitoring of CSF and blood, clinical and neurological evaluations, and magnetic resonance and ultrasound imaging of target organs demonstrated no toxicity related to treatment. AAV9-mediated gene transfer resulted in detection of sulfamidase activity in CSF throughout the study. Analysis at tissue level showed widespread sulfamidase expression and activity in the absence of histological findings in any region of encephalon, spinal cord, or dorsal root ganglia. Altogether, these results provide proof of durability of expression and long-term safety for intra-CSF delivery of AAV-based gene transfer vectors encoding therapeutic proteins to the CNS.

## Graphical abstract

**Figure:**
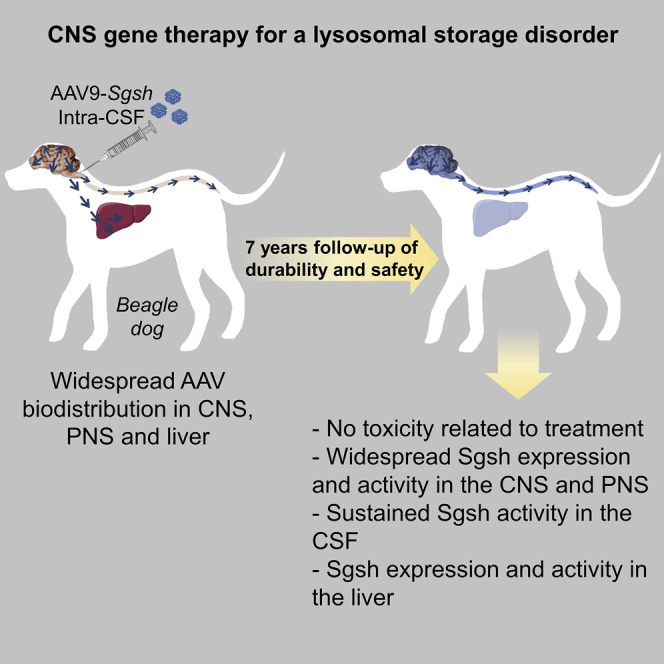

## Teaser

Seven-year follow-up of dogs after intra-CSF administration of AAV9-sulfamidase vectors results in detection of sulfamidase activity in CSF and widespread transgene expression in CNS, PNS, and liver, in the absence of any adverse events. Proof of durability and safety of this gene therapy support its clinical translation to treat CNS diseases.

## Introduction

The development of central nervous system (CNS)-targeted gene therapies for the treatment of neurological disorders is a very active field of research in both academia and industry.^1^, ^2^, ^3^, ^4^, ^5^, ^6^ CNS-targeted gene therapy is a modality of particular interest for those indications for which access of the target to the CNS from the circulation is limited. Our initial interest in the field of CNS gene therapy was for mucopolysaccharidosis type IIIA (MPSIIIA) or Sanfilippo syndrome type A. The Sanfilippo syndrome comprises a group of monogenic, autosomal recessive neurodegenerative disorders caused by mutations in enzymes involved in the stepwise degradation of the glycosaminoglycan (GAG) heparan sulfate (HS).^7^ As a consequence, undegraded HS accumulates in the lysosomes of cells, causing cell dysfunction and, eventually, cell death.^7^^,^^8^ Although the enzyme whose deficiency results in each subtype of Sanfilippo disease (types A–D) is different, all 4 subtypes share common pathophysiological underlying mechanisms as well as similar clinical signs and prognosis.^7^ MPSIII is considered mainly a neurodegenerative disease, with progressive cognitive and motor impairment that mainly correlate with cortical and cerebellar atrophy accompanied by a compensatory enlargement of the lateral ventricles in brain magnetic resonance imaging (MRI) images.^9^, ^10^, ^11^, ^12^, ^13^ There is, however, mild somatic involvement, with hepato- and splenomegaly, recurrent ear and upper respiratory tract infections, frequent diarrhea, and facial dysmorphisms. Disease progresses with age, leading to the death of affected individuals, generally in their second decade of life.^7^^,^^14^

In the case of lysosomal storage disorders (LSDs), most therapeutic strategies rely on the principle of cross-correction, where soluble lysosomal enzymes with mannose-6-phosphate (M6P) residues present in the extracellular compartment can be taken up by M6P receptor-mediated endocytosis into neighboring cells. Hence, transduction of large proportions of cells with gene therapies is not necessary to achieve full therapeutic efficacy.

Different ways of delivering genes to the CNS *in vivo* have been described so far. The simplest approach is based on the intravenous (i.v.) administration of adeno-associated viral vectors (AAVs) because of the ability of certain serotypes, such as serotype 9 (AAV9), to cross the blood-brain barrier (BBB) and transduce the CNS.^15^^,^^16^ This method is used to deliver Zolgensma, an FDA- and EMA-approved gene therapy that targets motor neurons of the spinal cord for the treatment of spinal muscular atrophy in pediatric patients.^17^ However, i.v. administration may not be the route of administration (ROA) of choice to reach the CNS in other neurodegenerative diseases that require widespread transduction of the CNS, or for the subset of patients that are seropositive for anti-AAV antibodies at high titers.^18^ Moreover, the high doses required for efficacy when using this ROA^8^^,^^19^, ^20^, ^21^ may trigger immunological responses against vector capsid.^22^ Clinical trials with systemically delivered AAVs are ongoing for MPSIIIA and MPSIIIB (NCT02716246, NCT04088734, NCT04360265, and NCT03315182).

Vectors encoding for lysosomal enzymes can also be delivered directly to the CNS by intraparenchymal injection or by administration into the cerebrospinal fluid (CSF). The first method, currently under clinical testing for MPSIIIA (NCT01474343, NCT02053064, and NCT03612869) and MPSIIIB (NCT03300453),^23^^,^^24^ requires a complex surgical procedure that involves the deposit of small volumes of AAV at two depths/site through 6–8 burr holes to cover a significant proportion of brain volume.^23^, ^24^, ^25^ However, the limited diffusion of the vector from the site of injection when delivered to most CNS regions other than the thalamus or striatum^26^, ^27^, ^28^, ^29^, ^30^ and the restricted number of injections that can be performed safely have resulted in limited proof of efficacy in clinical trials.^23^^,^^24^^,^^31^, ^32^, ^33^

Alternatively, we and others have demonstrated in several animal models the advantages of delivering vectors, in particular AAV9, directly into the CSF to achieve efficient and widespread CNS transduction.^34^, ^35^, ^36^, ^37^, ^38^, ^39^, ^40^, ^41^, ^42^ Whereas intraparenchymal administration results in uneven distribution of AAV vectors, with very high vector genomes at the point of administration quickly dropping with distance,^43^ intra-CSF delivery of AAV9 vectors ensures widespread, even distribution of transduced cells throughout the brain and spinal cord.^34^^,^^42^ Additional enhancement of therapeutic protein distribution is achieved for secretable transgenes, since transduced cells secrete proteins to the CSF efficiently.^34^^,^^35^^,^^39^^,^^44^ Furthermore, after intra-CSF administration, part of the vector passes from the CSF to the circulation and transduces the liver, which can secrete the therapeutic protein into the bloodstream.^34^, ^35^, ^36^^,^^39^^,^^44^^,^^45^

Using an AAV9 vector encoding sulfamidase (*Sgsh*), the enzyme deficient in MPSIIIA, we were the first to report whole-body correction of a LSD after delivery of AAVs to the CSF of mice.^34^ Although rodents provide an invaluable tool to perform proof-of-concept studies, anatomical/physiological species differences impose several limitations on non-clinical development. For example, the short lifespan of rodents precludes long-term evaluations of efficacy and safety. Therefore, it is ultimately data from larger animal species that best inform product behavior and distribution, in particular durability and long-term safety. In a previous short-term feasibility study in healthy dogs, we demonstrated that our approach was scalable to larger brains with a surgical procedure—intracerebroventricular delivery—that is standard practice in pediatric neurosurgery.^34^^,^^46^ In the present study, we report 7 years of follow-up of 3 healthy dogs injected in the CSF via cisterna magna with canine *Sgsh*-encoding AAV9 vectors at a clinically relevant dose. Our study demonstrates that the procedure is safe and results in high levels of SGSH activity in CSF detectable 7 years after a single vector delivery, in the absence of any adverse events. Transgene expression and activity were detectable within the CNS and the peripheral nervous system (PNS) at the end of the study. Vector persistence and transgene expression were also detectable in liver. To the best of our knowledge this is the longest safety and durability follow-up in a large animal species after CNS-directed gene transfer.

## Results

### General tolerability and safety of intra-CSF AAV9-sulfamidase administration

Three male healthy beagle dogs (dogs 1–3) were administered 2 × 10^13^ vg (2.78 × 10^11^ vg/ mL of dog brain) of AAV9 vectors carrying an optimized version of canine sulfamidase coding sequence under the control of the CAG promoter (AAV9-*Sgsh*) through cisterna magna injection.^34^ We previously reported the 3-month follow-up for these dogs.^34^ Here, we extend this original observation up to a total of 82 months (∼7 years).

Seven years after intra-CSF AAV9-*Sgsh* administration, treated dogs remained clinically well, with no signs of adverse events. CSF samples were tested regularly for elevations in white blood cell (WBC) counts or total protein (TP) levels as indicators of CNS inflammation. Red blood cells (RBCs) were counted in parallel to monitor for potential blood contamination during sample withdrawal. CSF WBC and TP levels remained within normal range throughout the study (Table 1). Only 1 in >50 CSF samples obtained from all 3 dogs showed WBC counts above the upper limit of normal (Table 1). This observation was made in a sample obtained from dog 1 51 months post-vector delivery, had only a 2× magnitude, and was accompanied by normal TP and no symptomatology (Table 1). Likewise, 3 samples presented TP levels slightly above the 25 mg/dL limit but showed no increase in WBCs, and dogs had no symptomatology (Table 1). Together with our previous monitoring throughout the first 3 months post-vector administration,^34^ the present observation extending up to 82 months post-test article delivery argue against the development of subclinical acute or chronic inflammatory processes in the CNS of the dogs injected with vectors encoding species-specific sulfamidase. This is in clear contrast with what has been observed previously in dogs and non-human primates (NHPs) administered vectors encoding human transgenes^34^^,^^47^ and underscores the importance of using species-specific transgenes for safety studies, particularly when evaluating long-term tolerance to a treatment.

**Table 1:** CSF cell counts and TP levels after intra-CSF delivery of 2 × 10^13^ vg of AAV9-*Sgsh* to healthy beagle dogs

Dog ID	Measure (reference values)	Months post-injection																
		4	6	8	10	12	16	20	24	28	32	38	46	51	61	65	72	82
Dog 1	RBC (0 cells)	0	0	0	0	0	0	0	0	0	0	0	0	0	0	0	0	0
	WBC (<5 cells/μL)	0	0	1	1	0	1	0	0	2	*NA*	1	0	*11*	0	0	4	0
	TP (<25 mg/dL)	19.3	24.8	22.7	20.2	19	18.2	20.2	20.6	22.9	21.4	21	23	21.5	*26.6*	*26*	20.4	22.2
Dog 2	RBC (0 cells)	59∗	0	0	0	0	0	0	0	0	*NA*	0	0	0	0	0	0	0
	WBC (<5 cells/μL)	1∗	1	4	0	5	1	1	1	1	*NA*	0	0	1	5	0	0	0
	TP (<25 mg/dL)	19.4∗	22.8	21.6	19.1	21.1	22.6	23.2	20.9	21.9	*NA*	20.5	22.2	21.3	21.2	24	20.7	*25.9*
Dog 3	RBC (0 cells)	0	0	0	0	0	0	0	0	0	0	0	0	0	0	0	0	0
	WBC (<5 cells/μL)	0	1	2	0	1	1	1	0	1	*NA*	1	0	0	1	0	0	0
	TP (<25 mg/dL)	19.6	18.9	19.4	21.7	18.9	22.6	21.7	24.6	20.5	17.4	20.1	21.3	20.9	21.8	24.2	21	20.9

∗ Sample contaminated with blood. White blood cell (WBC) and total protein (TP) values were corrected taking into account blood contamination. RBC, red blood cell; NA, sample not available. The values outside the reference interval are italicized.

To further assess potential toxicities, blood tests were regularly performed. Although several isolated deviations in clinical chemistry or hematological parameters were observed in different samples from all 3 dogs throughout the 7-year follow-up, likely reflecting suboptimal sample quality, abnormalities were considered either to be of clinically irrelevant magnitude or not to follow a pattern compatible with test article- or procedure-related toxicities, which agreed with the general health status of the animals (Tables S1 and S2).

### CNS and liver imaging and functional neurological evaluation

As part of the long-term safety evaluation, MRI scans of the encephalon and the spinal cord, as well as abdominal ultrasounds, were obtained at different time points after vector administration in all injected dogs.

**Figure 1:**
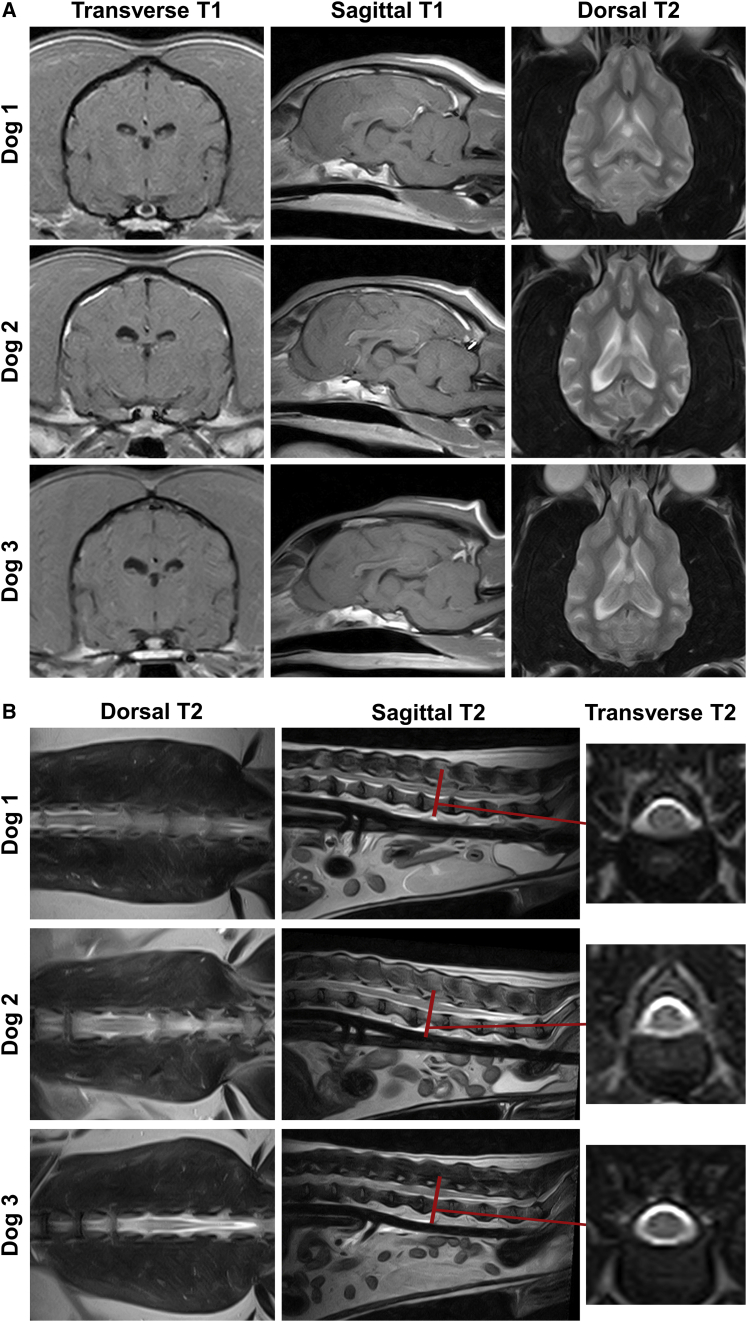
Preserved structure of the encephalon and the spinal cord after intra-CSF AAV9-*Sgsh* delivery Adult beagle dogs (dogs 1–3) received a dose of 2 × 10^13^ vg of AAV9 vectors encoding canine sulfamidase through intracisternal administration. (A) Representative images of the magnetic resonance imaging (MRI) of the encephalon of dogs 1–3 performed 82 months after vector delivery. Scans were performed with a 0.2-T permanent open magnet system. Left and middle: transverse and sagittal T1-weighted images, respectively, obtained after intravenous administration of a gadolinium-containing contrast agent. Right: dorsal T2-weighted images obtained prior to contrast injection. No abnormalities were observed in the encephalon of any of the AAV9-*Sgsh*-injected dogs in any of the MRI sequences. (B) Representative images of the MRI analysis of the spinal cord performed 82 months after vector delivery, focused on the lumbosacral intumescence and the cauda equina, as previous work had determined that these were the portions of the spinal cord most efficiently transduced after intra-CSF administration of AAV9 vectors to dogs. A 0.4-T scanner was used to obtain dorsal (left), sagittal (middle), and L_2–_L_3_ transverse (right) T2-weighted images. Similar to the encephalon, no abnormalities could be identified in any of the dogs after systematic analysis of all the images obtained.

Brain MRI scans were performed 34 (Figure S1) and 82 (Figure 1A) months after vector delivery, in which dorsal T2-weighted images and transverse and sagittal T1-weighted images after intravenous administration of the contrast agent gadolinium were acquired for all dogs. Systematic analysis of the scans revealed no abnormalities in the encephalon of any of the AAV-injected dogs in any of the MRI sequences studied. Scans showed no evidence of mass effect, asymmetries, or significant alterations in signal intensity at any of the time points studied (Figure 1A; Figure S1) and were indistinguishable from those of healthy uninjected dogs (Figure S2). After administration of the contrast, no signs of enhancement could be observed in any region of the encephalon or the meninges in any of the dogs (Figure 1A; Figures S1 and S2).

In a previous study, we observed that after delivery of AAV9 vectors to the CSF of dogs through either cisterna magna or lateral ventricle injection, the lumbosacral intumescence and the cauda equina were the sections of the spinal cord with the highest levels of transgene expression.^34^ Hence, we focused on these regions of the spinal cord for the evaluation through MRI. Sagittal and transverse T1-weighted (pre- and post-contrast administration) and dorsal, sagittal, and transverse T2-weighted scans were obtained for all dogs 60 and 82 months after vector delivery; representative images are shown in Figure S3 and Figure 1B. Similar to the encephalon, no abnormalities could be identified in any of the dogs after systematic analysis of all the images obtained.

In addition, comprehensive abdominal ultrasounds were performed 34 months after intra-CSF AAV9-*Sgsh* vector injection. Special focus was placed on the analysis of the liver, the main peripheral organ transduced by AAV9 vectors after delivery to the CSF of small and large animal models.^34^, ^35^, ^36^, ^37^^,^^39^^,^^40^^,^^45^ Liver parenchyma was homogeneous and presented normal echogenicity in the 3 dogs. Livers were of normal size and echostructure, and there was no evidence of alterations in hepatic vascularization or the blood flow through the portal vein.

All animals remained clinically well throughout the whole follow-up period. To further evaluate the possibility of toxicities specifically in the nervous system, full neurological evaluations were performed in all dogs by a diplomate veterinary neurologist 52 and 82 months after AAV9-*Sgsh* delivery to the CSF (Table S3). The neurological examinations included observation of the mental status, posture, and gait as well as evaluation of postural reactions, spinal reflexes, and cranial nerves and sensory evaluation (pain sensation and detection of areas of hyperesthesia) (Table S3). In consonance with the MRI results, no signs of diffuse or focal lesions were observed in any of the AAV9-injected dogs.

### Time course of sulfamidase activity in the CSF and serum

**Figure 2:**
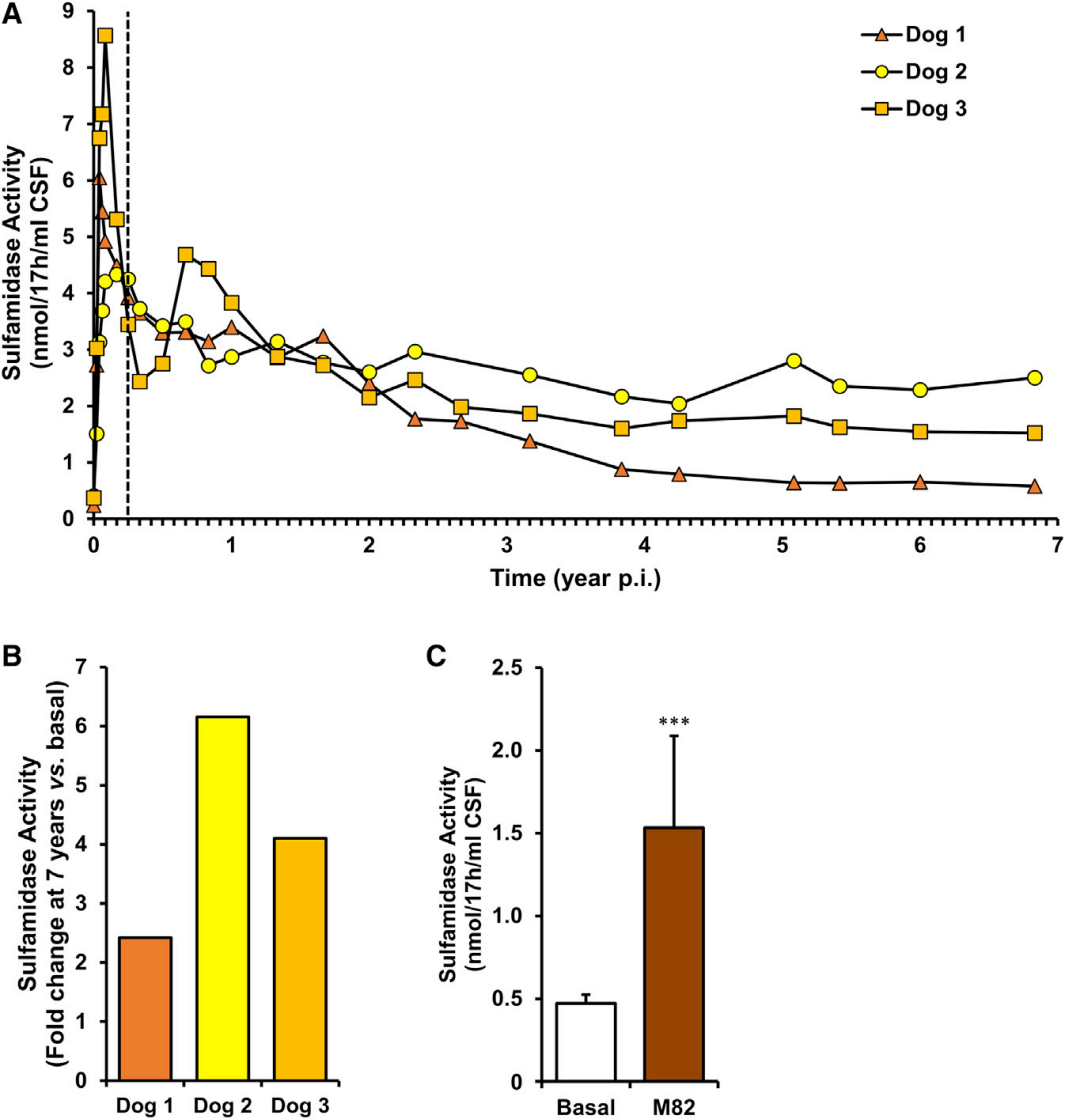
Follow-up of sulfamidase activity in the CSF of AAV9-*Sgsh*-injected dogs (A) CSF samples were withdrawn regularly from dogs 1–3 for a period of 7 years and assayed for sulfamidase activity. All AAV9-injected dogs showed a substantial increase in SGSH activity in the CSF over baseline levels, and activity remained high for the whole of the follow-up period. As a reference, the basal CSF sulfamidase activity quantified in CSF samples from 15 untreated healthy beagle dogs was 0.47 ± 0.05 nmol/17 h/mL of CSF. p.i., post injection. The vertical dashed line indicates the first 3 months of treatment. (B) Fold-change increase over basal levels in CSF sulfamidase activity at the end of the study (82 months post-injection) represented for each dog. Seven years after treatment, CSF sulfamidase activity remained at least 2.4-fold higher than at baseline in all AAV9-injected dogs shown. (C) Sulfamidase activity in the CSF at the basal time point from 15 untreated healthy beagle dogs and at the end of the study (M82, 82 months post-injection) from the three AAV9-injected dogs. Results are shown as mean ± SEM. A two-tailed t test was used for statistical comparison of CSF sulfamidase activity at the endpoint (82 months post-injection) with the basal CSF sulfamidase activity. ^∗∗∗^p < 0.001.

As previously reported,^16^ a clear increase in sulfamidase activity over basal levels was detectable in the CSF of all 3 dogs as soon as 1 week after vector administration. The time to peak activity after vector delivery varied depending on the dog. Dogs 1 and 3 showed a rapid raise in enzymatic activity, which peaked 2 and 4 weeks post-injection, respectively (Figure 2A).^34^ Dog 2 showed a slower kinetics of transgene expression and had peak values of SGSH activity at 1–3 months post injection that were lower than those observed in dogs 1 and 3 (Figure 2A).^34^ At the end of this first phase of the study (90 days) dogs had an average sulfamidase activity in the CSF 8.2-fold higher than the average basal levels. By 1 year post injection all dogs had reached similar levels of SGSH activity in the CSF (3.40, 2.87, and 3.83 nmol/17 h/mL CSF for dogs 1, 2, and 3, respectively), which was on average 7.1-fold higher than that documented in uninjected dogs (0.47 ± 0.05 nmol/17 h/mL, n = 15 dogs) (Figure 2A). After the first 24 months, CSF SGSH activity remained fairly stable in dogs 2 and 3 for the whole of the 7-year follow-up period. By year 7, dogs 2 and 3 had levels of SGSH activity in the CSF that were 6.2-fold and 4.1-fold higher than baseline (Figures 2A and 2B). In contrast, in dog 1 CSF SGSH activity progressively decreased with time, stabilized around 4 years post injection, and was 2.4-fold higher than baseline at the end of the follow-up period (Figures 2A and 2B). The average value of CSF sulfamidase activity for the 3 dogs 7 years post vector delivery is statistically highly significant (p value = 0.0005) compared to the average value measured in the CSF of 15 untreated dogs (∼3-fold increase) (Figure 2C). To the best of our knowledge, these expression data constitute the longest follow-up of transgene expression after delivery of AAVs to the CNS of a large animal.

We and others have previously reported that upon administration of AAV9 vectors to the CSF of mice, cats, dogs, or NHPs, a portion of the vector passes to the circulation and transduces the liver, which can become a source of circulating enzyme.^34^, ^35^, ^36^, ^37^, ^38^, ^39^, ^40^, ^41^, ^42^^,^^45^ However, no obvious increase in sulfamidase activity was detected in the serum of any of the animals injected with AAV9-*Sgsh*, likely because of the high basal level of sulfamidase activity in the serum of healthy dogs.

### Post-mortem analysis of vector biodistribution and transgene expression in the CNS and DRG

**Figure 3:**
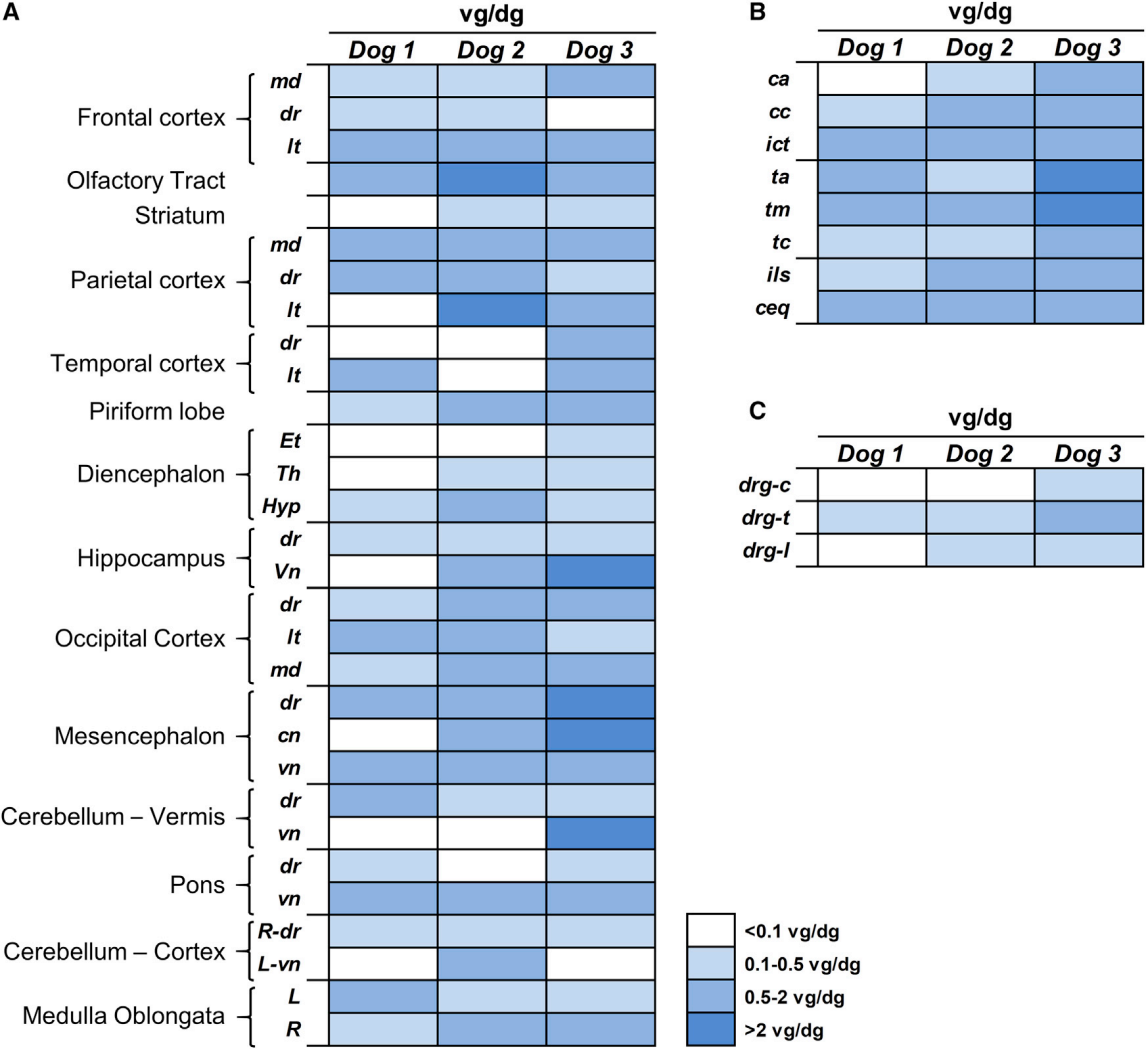
Widespread detection of vectors within the CNS and PNS ganglia Vector gene copy number was analyzed in tissue punches from multiple regions of the encephalon (A), spinal cord (B), and dorsal root ganglia (C) obtained during necropsy of dogs 1–3. md, medial; dr, dorsal; lt, lateral; vn, ventral; cn, central; R, right; L, left; ca, cervical anterior; cc, cervicocaudal; ict, cervicothoracic intumescence; ta, thoracic anterior; tm, thoracic medial; tc, thoracic caudal; ils, lumbosacral intumescence; ceq, cauda equina; drg, dorsal root ganglia; c, cervical; t, thoracic; l, lumbar; Et, epithalamus; Th, thalamus; Hyp, hypothalamus. vg/dg, vector genomes/diploid genome.

At the end of the in-life portion of the study, samples were collected from different areas of the encephalon (n = 30/dog), spinal cord (n = 8/dog, covering cervical, dorsal, and lumbar portions), and dorsal root ganglia (DRGs) (cervical, dorsal, lumbar) for quantification of vector genome copy number (VGCN) and sulfamidase mRNA levels. One non-injected dog was used as negative control. Similar to what was previously observed in shorter (3 month) studies in dogs,^34^ quantification of VGCN demonstrated widespread distribution of the vector within the CNS and PNS ganglia (Figure 3**)**. In the encephalon, 83% and 93% of samples from dogs 2 and 3 tested positive for AAV9-*Sgsh* vector genomes (Figure 3A). The percentage was slightly lower for dog 1, in which 70% of samples had copy numbers above 0.1 vector genomes/diploid genome (vg/dg) (Figure 3A). In the spinal cord, values were more similar among all 3 dogs; we failed to detect vector genomes in only 1 of 10 spinal cord samples from dog 1 (Figure 3B). DRGs showed relatively low VGCN, and for dog 1, 2 out of 3 samples tested negative, although this observation could be limited by sampling, as the few DRGs collected had to be distributed among the different analyses (Figure 3C).

**Figure 4:**
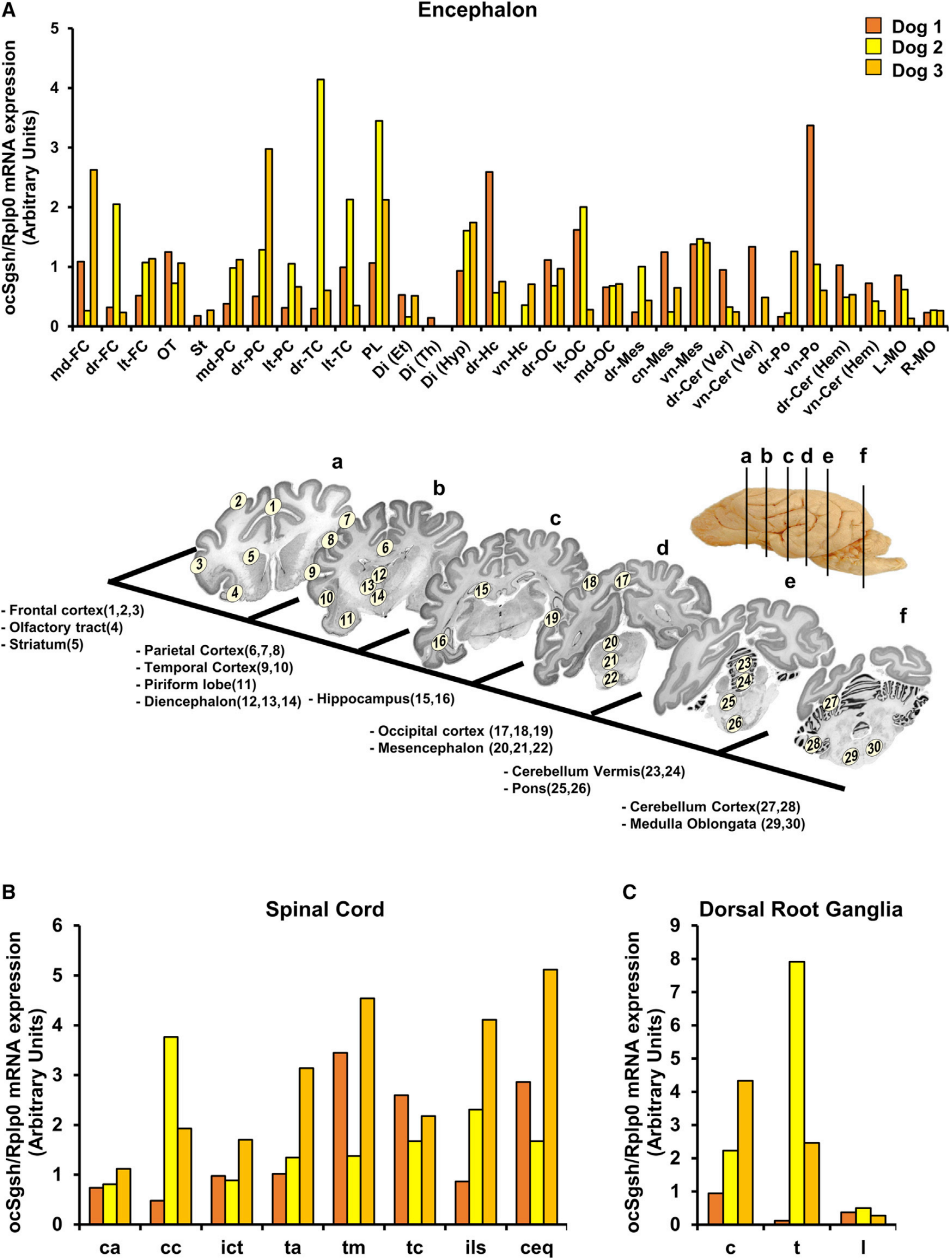
Persistent vector-derived sulfamidase expression in the CNS and PNS ganglia Optimized canine sulfamidase mRNA expression was measured in tissue punches obtained from multiple regions of the encephalon (A), spinal cord (B), and dorsal root ganglia (C) obtained from dogs 1–3 7 years after vector injection. In (A), histograms depict the relative sulfamidase expression (normalized to housekeeping gene) in all areas of the encephalon analyzed, ordered as per their location in the left hemisphere, as indicated in the schematic representation included below the histogram. md-FC, medial frontal cortex; dr-FC, dorsal frontal cortex; lt-FC, lateral frontal cortex; OT, olfactory tract; St, striatum; md-PC, medial parietal cortex; dr-PC, dorsal parietal cortex; lt-PC, lateral parietal cortex; dr-TC, dorsal temporal cortex; lt-TC, lateral temporal cortex; PL, piriform lobe; Di (Et), diencephalon (epithalamus); Di (Th), diencephalon (thalamus); Di (Hyp), diencephalon (hypothalamus); dr-Hc, dorsal hippocampus; vn-Hc, ventral hippocampus; dr-OC, dorsal occipital cortex; lt-OC, lateral occipital cortex; md-OC, medial occipital cortex; dr-Mes, dorsal mesencephalon; cn-Mes, central mesencephalon; vn-Mes, ventral mesencephalon; dr-Cer (Ver), dorsal cerebellum (vermis); vn-Cer (Ver), ventral cerebellum (vermis); dr-Po, dorsal pons; vn-Po, ventral pons; dr-Cer (Hem), dorsal cerebellum cortex; vn-Cer (Hem), ventral cerebellum cortex; L-MO, left medulla oblongata; R-MO, right medulla oblongata; ca, cervical anterior; cc, cervicocaudal; ict, cervicothoracic intumescence; ta, thoracic anterior; tm, thoracic medial; tc, thoracic caudal; ils, lumbosacral intumescence; ceq, cauda equina; c, cervical; t, thoracic; l, lumbar.

Codon optimization of the transgene allowed for differentiation of vector-derived sulfamidase mRNA from endogenous transcripts. Despite the slight differences in VGCN, all 3 dogs showed similar levels and distribution of sulfamidase expression in the encephalon, with at least 90% of positivity among all samples analyzed (Figure 4A). The regions positive for *Sgsh* expression covered the whole volume of the encephalon, from areas close to the cisterna magna (site of injection into the CSF), such as cerebellum, pons, and medulla oblongata, to areas distant from the injection site, such as frontal cortex and olfactory tract (Figure 4A). Moreover, sulfamidase expression was detected in deeper areas of the brain, consistent with vector distribution through CSF-mediated diffusion,^34^ although some of these deep brain structures, such as the striatum, the epithalamus, and the thalamus, had very low to no detectable mRNA levels (Figure 4A). Expression of AAV9-*Sgsh* vectors also persisted throughout the years in the spinal cord of all 3 dogs, from the cervical region down to the cauda equina, on average at levels similar to those documented in the brain of the respective animals except for dog 3, in which they seemed to be slightly higher (Figure 4B). DRGs were also positive for transgene expression (Figure 4C). Although some of the DRG samples from dog 1 had tested negative for vector genomes, expression of codon-optimized canine sulfamidase was detectable at low levels in all samples. Overall, these results showed persistent, widespread AAV9 vector distribution and transgene expression within the CNS and PNS ganglia of a large animal up to 7 years after delivery to the CSF.

### Distribution of active sulfamidase within CNS

**Figure 5:**
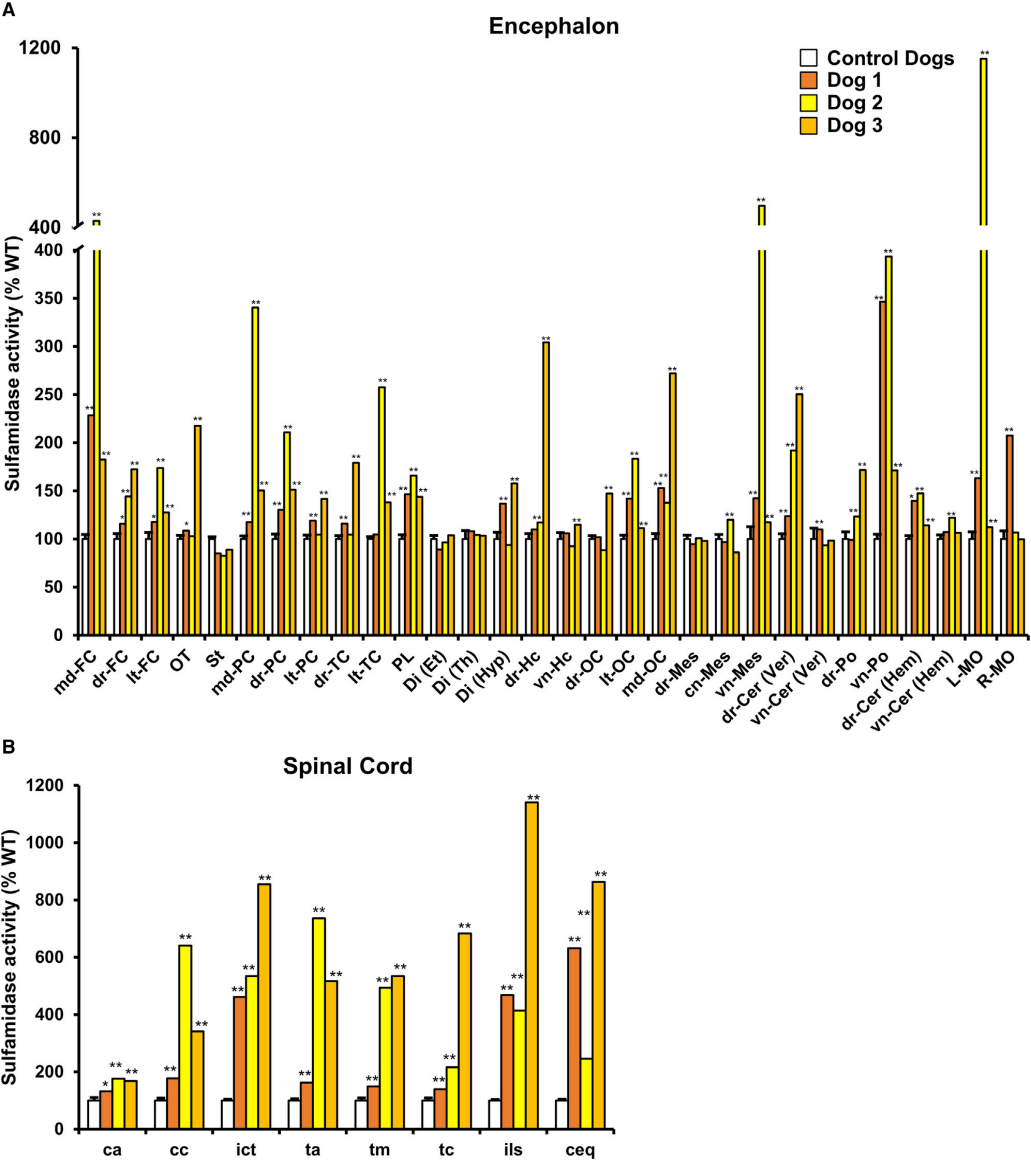
Tissue sulfamidase activity levels in the CNS Quantification of sulfamidase activity in the encephalon (A) and spinal cord (B) of dogs administered AAV9-*Sgsh*. Sulfamidase activity is represented as % of wild-type (WT) activity for each region. To establish this WT reference value, sulfamidase activity was quantified in each CNS area in samples obtained from 15 untreated, healthy beagle dogs (n = 10–15/region). WT reference values are shown as mean ± SEM. ^∗^The value of injected dogs is out of the 95% confidence interval (CI) (versus WT); ^∗∗^the value of injected dogs is out of the 99% CI (versus WT). md-FC, medial frontal cortex; dr-FC, dorsal frontal cortex; lt-FC, lateral frontal cortex; OT, olfactory tract; St, striatum; md-PC, medial parietal cortex; dr-PC, dorsal parietal cortex; lt-PC, lateral parietal cortex; dr-TC, dorsal temporal cortex; lt-TC, lateral temporal cortex; PL, piriform lobe; Di (Et), diencephalon (epithalamus); Di (Th), diencephalon (thalamus); Di (Hyp), diencephalon (hypothalamus); dr-Hc, dorsal hippocampus; vn-Hc, ventral hippocampus; dr-OC, dorsal occipital cortex; lt-OC, lateral occipital cortex; md-OC, medial occipital cortex; dr-Mes, dorsal mesencephalon; cn-Mes, central mesencephalon; vn-Mes, ventral mesencephalon; dr-Cer (Ver), dorsal cerebellum (vermis); vn-Cer (Ver), ventral cerebellum (vermis); dr-Po, dorsal pons; vn-Po, ventral pons; dr-Cer (Hem), dorsal cerebellum cortex; vn-Cer (Hem), ventral cerebellum cortex; L-MO, left medulla oblongata; R-MO, right medulla oblongata; ca, cervical anterior; cc, cervicocaudal; ict, cervicothoracic intumescence; ta, thoracic anterior; tm, thoracic medial; tc, thoracic caudal; ils, lumbosacral intumescence; ceq, cauda equina.

To fully map distribution of active sulfamidase in the CNS of dogs 7 years after gene transfer, we measured SGSH activity in tissue extracts obtained from the encephalon (n = 30/dog) and the spinal cord (n = 8/dog; covering cervical, dorsal, and lumbar portions). The enzymatic assay used cannot distinguish endogenous from vector-derived sulfamidase. Contrary to CSF, where background SGSH activity is very low, allowing for sensitive monitoring of transgene expression, tissue SGSH activity in the CNS is relatively high, which lowers the sensitivity of the assay to detect increments over baseline. The brain and spinal cord of 15 control animals not injected with the SGSH vectors were used to obtain reference values for endogenous activity for each region analyzed, with a sample size of at least 10 samples per region, totaling >400 measurements. In agreement with biodistribution and transgene expression data, increases in sulfamidase activity over the levels detected in control dogs were documented in >70% of the encephalon and spinal cord samples obtained from each of the injected dogs (Figure 5A). The magnitude of the increase over control levels varied significantly depending on the region analyzed and was frequently—but not always—consistent across animals. For example, in all 3 dogs, the increments observed in the meridial portion of the frontal cortex were significant, whereas in the diencephalon they were very modest (Figure 5A). In contrast, the variability was higher in pons or medulla oblongata samples, in which values could vary by a factor of 10× among different animals. On the other hand, spinal cord samples showed consistently high levels of SGSH activity in all animals (Figure 5B). For some of the dogs, the levels of SGSH activity documented in the spinal cord extracts represented the highest of all the levels measured in that dog.

### Histological analysis of the CNS and DRG

At sacrifice, we also collected encephalon, spinal cord, and DRG samples for detailed histological analysis. Sections obtained from different regions of the CNS in which we had documented high transgene expression, including frontal, parietal, and occipital cortices, hippocampus, cerebellum, and spinal cord, were analyzed in detail after hematoxylin and eosin (H&E) and Nissl staining and immunostaining against GFAP and Iba1. Tissues showed normal structure, and no histopathological observations were made that would be suggestive of long-term product-related toxicities (Figures S4–S7).

**Figure 6:**
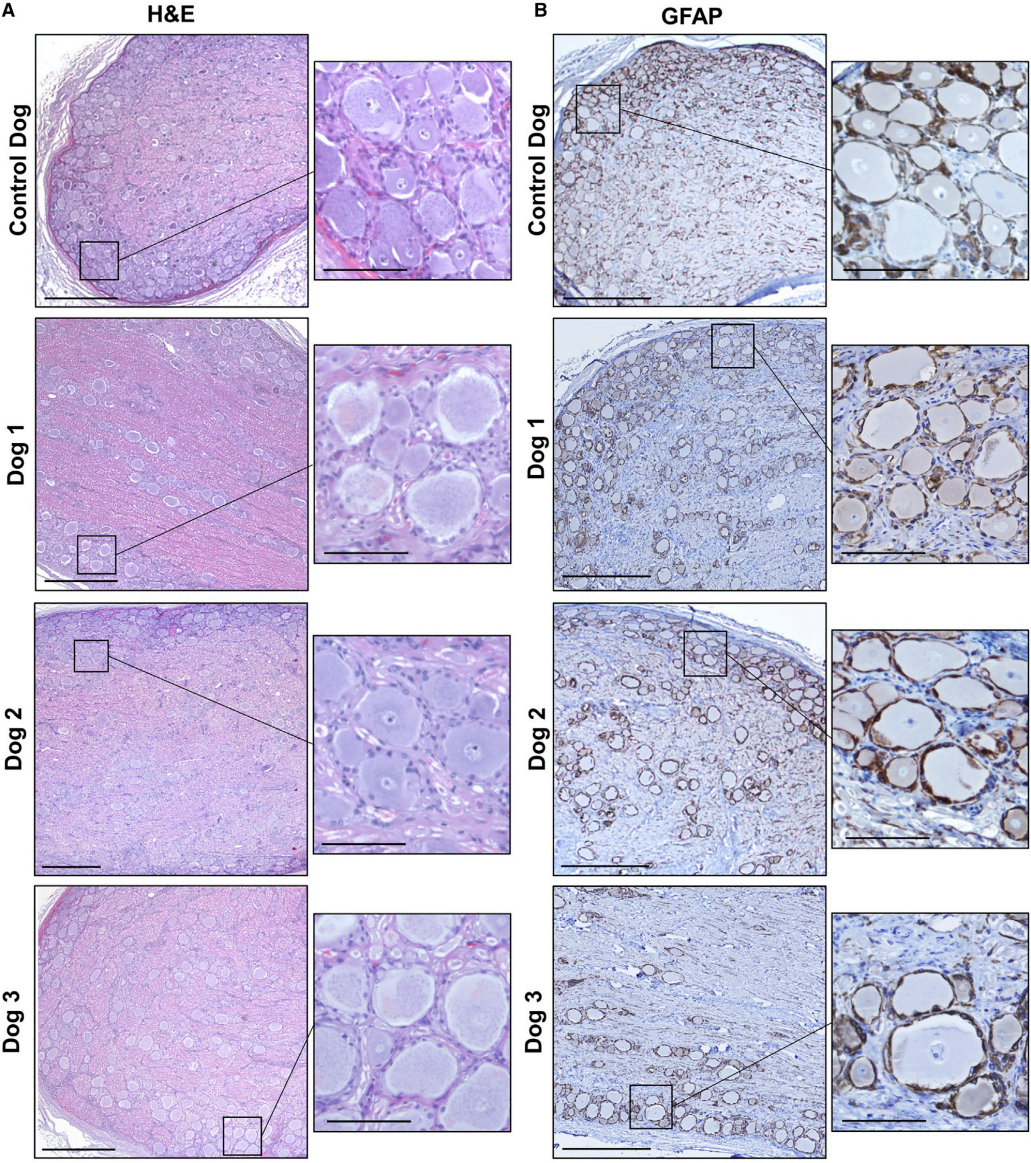
Histopathological analysis of PNS dorsal root ganglia Representative images corresponding to lumbar DRGs obtained from dogs 1–3 7 years post-vector administration and from a control, uninjected dog after H&E staining (A) and immunohistochemistry for astrocyte marker GFAP (B). Scale bars, 500 μm; insets, 100 μm.

We paid special attention to DRGs, given the recent reports that have uncovered histopathological findings in the DRGs of primates after intravenous or intra-CSF delivery of high doses of AAV vectors.^47^, ^48^, ^49^, ^50^ DRG samples were taken from each of the AAV-injected dogs at three levels: cervical, thoracic, and lumbar. To make the assessment through the whole dimension of the extracted DRG, several sections were obtained at 3 different levels of each ganglion. At each level, the first section was used for H&E staining and the consecutive sections were used for immunohistochemistry against GFAP and Iba1, making a total number of 9 sections analyzed per DRG, and were compared to the findings in a control, non-injected dog by a diplomate veterinary pathologist. In all H&E sections from all 3 AAV-injected dogs, neuronal cell bodies of normal appearance and distribution and in normal numbers were observed, with no evidence of neuronal fragmentation or neuronophagia (Figure 6; Figure S8). GFAP reactivity was observed in satellite cells and Schwann cells, and Iba1 reactivity was very weak and limited to mononuclear cells scattered among the neuronal bodies and occasionally in cells grouped in nodules of mononuclear cells, which was a rare finding (Figure 6; Figure S8). Figure 6 shows representative images of a lumbar DRG obtained from dog 2; DRG toxicities have been described to affect mostly the lumbar region and depend on transduction levels,^48^ and dog 2 was the dog with the highest levels of SGSH activity in CSF.

To complete the analysis, we studied spinal cord samples corresponding to the same regions (cervical, thoracic, and lumbar) with the same histological tools. Tissue architecture was preserved in all injected dogs; the distribution of GFAP reactivity was homogeneous, with an abundance of astrocytes but with no sign of gliosis. Iba1 staining was weak and was also homogeneously distributed. Altogether, the samples were considered to have no significant structural changes, and the analysis discarded the presence of a chronic inflammatory process.

### Transgene expression and activity in the liver

After vector administration into the CSF, AAV9 vectors have been systematically detected in the liver of mice, dogs, and NHPs,^34^^,^^35^^,^^39^^,^^45^^,^^47^^,^^51^ indicating that intra-CSF administration of AAV9 vectors could potentially transduce the liver, an organ known to efficiently produce and secrete proteins to the bloodstream.^34^^,^^35^^,^^39^^,^^45^ Percutaneous ultrasound-guided liver biopsies were obtained from the 3 dogs injected with AAV9-*Sgsh* 48 and 54 months after vector administration. Two samples, from the left median and left lateral lobes, were obtained from each animal at each biopsy time point (total of 4 biopsies per animal). One non-injected dog and one dog injected in the CSF with AAV9-GFP vectors at the same dose as in the present study were used as negative controls.

The results of the analysis of transgene expression by quantitative real-time PCR in these biopsies were variable. Dog 2 had the highest levels of expression in both lobes, dog 3 had detectable expression at intermediate levels in the left lateral lobe, and dog 1 showed a transcript signal in the same range as negative controls (Table S4). The corresponding quantification of VGCN in another portion of the same biopsies paralleled the findings of transcript quantification. Whereas no vector genomes could be detected in the median or lateral lobes of the liver of dog 1, dog 2 showed 1–2 vector genome copies/cell in each of the 4 different biopsies analyzed, and dog 3 presented detectable vector genomes in both lobes, albeit at lower copy numbers than dog 2 (Table S4). Altogether, these results suggest that the differences in peripheral sulfamidase expression are due to different degrees of hepatocyte transduction and not to the potential silencing of the transgene.

**Figure 7:**
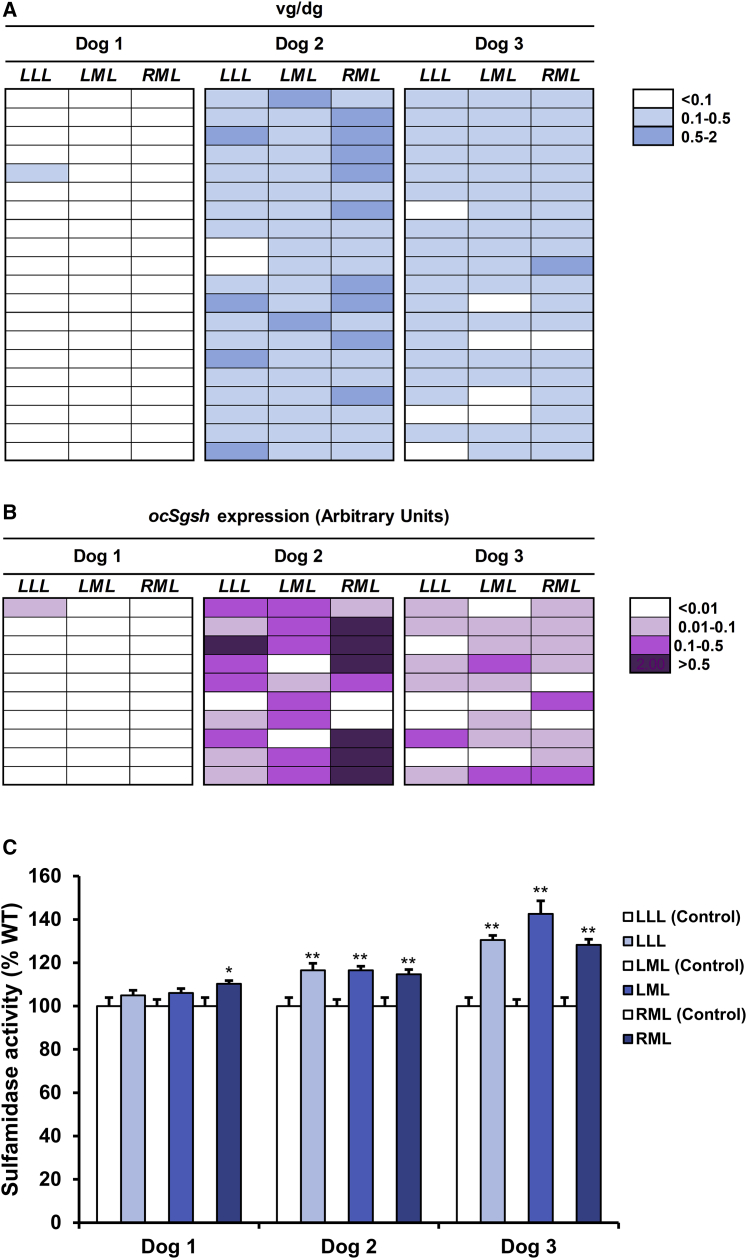
Dissemination and persistence of AAV9-*Sgsh* vectors in the liver after intra-CSF delivery to dogs (A) In each dog, vector gene copy number was analyzed in 20 samples per lobe to fully map AAV9 biodistribution in the liver. vg/dg, vector genome/diploid genome. (B) Optimized canine sulfamidase mRNA was quantified in 10 samples per liver lobe in each dog and referenced to the expression of the housekeeping gene *Rplp0*. (C) Sulfamidase activity was determined in 20 samples per liver lobe per dog and reported as % of wild-type (WT) activity for that lobe. To obtain the WT reference value, endogenous sulfamidase activity was quantified in 1 sample per liver lobe obtained from 15 untreated, healthy beagle dogs (left lateral lobe = 6.61 ± 0.26 nmol/17 h/mg protein; left medial lobe = 6.11 ± 0.19 nmol/17 h/mg protein; and right medial lobe = 6.49 ± 0.26 nmol/17 h/mg protein). Results are shown as mean ± SEM.^∗^The mean value in injected dogs is out of the 95% confidence interval (CI) (versus WT); ^∗∗^the mean value of injected dogs is out of the 99% CI (versus WT).

At the end of the study, vector biodistribution was evaluated in additional liver samples obtained during necropsy. To get more representative data than those provided by biopsies, 20 evenly distributed samples per lobe were collected from each dog (approximately n = 60 samples/liver). One non-injected dog was used as negative control. In agreement with the observations made in biopsy samples, vector genomes were only detected in the livers of dogs 2 and 3; for dog 1 all samples except one were negative (Figure 7A). Remarkably, and with the caveat that samples were analyzed with a different quantitative real-time PCR assay, in all 3 dogs VGCN values were very similar in biopsy and necropsy samples obtained 3 years apart. AAV9-derived transgene expression analyzed by qRT-PCR fully correlated with VGCN data; the highest levels of transgene expression were detected in dog 2, which had the highest VGCN, followed by dog 3, with almost no expression detectable in dog 1 (Figure 7B). Finally, sulfamidase activity was evaluated in an equal number of liver samples (20 per lobe) and averaged per lobe. Increases over baseline levels, established for each lobe averaging samples from 15 control dogs, were documented in dogs 2 and 3, at levels that did not correlate with VGCN and expression data, which likely reflected the limitations of the SGSH activity assay in samples with high endogenous activity (Figure 7C).

At sacrifice, we also collected liver samples for detailed histological analysis. Liver showed normal structure, and no histopathological alterations were observed that would be suggestive of long-term product-related toxicities (Figure S9).

### Baseline anti-AAV9 immunity and response to vector exposure

In an attempt to better understand the differences in transduction observed across the dogs, we did a longitudinal evaluation of neutralizing antibodies (NAbs) against AAV9 in paired CSF and serum samples. At baseline, none of the dogs had anti-AAV9 NAbs in CSF (Table S5). Two of the dogs (dogs 2 and 3) were seronegative for circulating NAbs, but dog 1 did have anti-AAV9 NAbs at low titers (1:5–1:10) (Table S5). After vector administration, NAbs rose significantly in serum, with the highest values observed in day 28 samples, and decreased thereafter, stabilizing at relatively modest titers years after exposure to the vector (Table S5). Interestingly, the rise in circulating NAbs was most significant in the animal that was seropositive at baseline. The change in levels of CSF NAbs followed a kinetics similar to that in serum, albeit remaining always at very low titers (1:5–1:80).

## Discussion

Genetic diseases that affect the brain constitute important unmet medical needs. LSDs with neurological involvement, in particular, represent a complex therapeutic challenge, as the enzyme replacement therapies (ERTs) that might alleviate peripheral disease are ineffective to treat the CNS because of the presence of the BBB. The option of providing the enzyme directly to the CNS has been tested clinically, although the few trials performed so far have revealed the shortcomings associated with the implantation of the intrathecal delivery devices that are required for periodic infusion of proteins to the CSF.^52^^,^^53^ CNS-targeted gene therapy represents a promising therapeutic option for many of these diseases. Successful transfer to the clinic of proof-of-concept studies performed in rodents relies heavily on the demonstration of feasibility and safety in large animal models that are anatomically and physiologically closer to the intended target population. The volume of the adult mouse brain is on average ∼0.45 cm^3^,^54^, ^55^, ^56^, ^57^ ∼2,800-fold smaller than that of a human (1,328 and 1,205 cm^3^ for male and females, respectively, at age 4 years 9 months to 18 years^58^). The (beagle) dog, with a brain volume in an adult animal of ∼70 cm^3^ and a lifespan of ∼13 years (S. Yu, 2010, Fed. Am. Soc. Exp. Biol., abstract), offers a better model to address clinically relevant questions such as distribution of the therapeutic vector or durability of transgene expression.

Previous studies have unequivocally established the potential of AAV vectors to correct CNS disease with a variety of gene transfer methodologies.^1^ Some of these have also demonstrated feasibility and tolerability in large animal models. Few of them, however, have addressed the long-term safety and durability of effect of the different approaches tested. Here, we report what to the best of our knowledge is the longest follow-up (almost 7 years) after gene transfer to the CNS of a large animal. We demonstrate stable, multi-year production of active sulfamidase, the enzyme defective in MPSIIIA, in the absence of any signs of acute or chronic toxicity.

Our approach to MPSs is based on the delivery of vectors encoding the missing enzyme into the CSF. The rationale behind this choice is that the use of this fluid as the route of delivery allows for better distribution of the vectors within the CNS, the main target compartment for this disease. Although other serotypes have been delivered to the CSF,^59^^,^^60^ we and others have demonstrated that AAV9 mediates efficient and widespread transduction of the encephalon and the spinal cord.^34^^,^^36^^,^^38^^,^^40^ If the therapeutic transgene is secretable, the protein can generally be detected in the CSF, and distribution by the CSF can potentially enhance the therapeutic effect. In this regard, intrathecal ERT has offered valuable evidence of the distribution of lysosomal enzymes in the CNS via the CSF.^61^^,^^62^

In a previous report, we demonstrated that upon delivery of AAV9 vectors encoding human sulfamidase to the CSF of dogs at the vector dose deemed therapeutic in MPSIIIA mice, activity of the enzyme in the CSF peaked within 2–3 weeks.^34^ This increment was, however, accompanied by signs of CNS inflammation, such as a rise in TP levels and WBC counts in the CSF of injected animals, and subsequently the transgene activity in the CSF dropped. Despite the inflammatory reaction, sulfamidase activity in the CSF always remained above basal levels, and at termination sulfamidase expression was detectable within different areas of the CNS, as well as in the PNS and somatic tissues.^34^ These observations prompted us to use the same vector backbone but replace the human sulfamidase coding sequence by the species-specific open reading frame (ORF). This approach allowed us to analyze tolerability and durability of expression in the absence of confounding variables, such as immune responses or use of intense immunosuppressive regimes. Additionally, MPSIIIA patients have shown that >98% of subjects are carriers of at least one allele corresponding to a missense mutation.^7^ Thus, most patients are expected to have some degree of tolerance to sulfamidase, as opposed to dogs to the human protein, many epitopes of which could constitute neoantigens. Therefore, we consider healthy beagle dogs injected with canine sulfamidase to be a better predictor of possible outcomes than beagle dogs treated with human sulfamidase.

AAV vectors encoding the canine sulfamidase were injected into the CSF at the same dose as that of the human sulfamidase, and animals were followed for almost 7 years. The initial kinetics of canine sulfamidase expression was similar to that of the human transgene, with an initial peak within 2–4 weeks post-administration. In contrast to the study that used human sulfamidase as a transgene, the activity of sulfamidase in the CSF of dogs injected with the canine sulfamidase-bearing vectors did not drop to pre-treatment levels after a few weeks.^34^ On the contrary, CSF sulfamidase activity remained elevated for several years, at steady-state levels that were at least ∼2.4-fold higher than those detected at baseline. A similar profile of transgene expression, in which the levels of transgene protein peak a few weeks after vector administration before stabilizing at steady-state levels a few weeks or months later in complete absence of any sign of toxicity, has been observed for other secretable proteins when expressed from ubiquitous or tissue-specific promoters in a variety of organs.^34^^,^^39^^,^^40^^,^^47^^,^^63^, ^64^, ^65^, ^66^ The mechanisms underlying this profile of expression are not fully understood, but failure to stabilize all of the vector genomes present in the initial load into stable episomes has been postulated to play a role.^67^^,^^68^ It is worth noting that we used healthy dogs for this study, with normal production of sulfamidase. Although for dogs 2 and 3 SGSH activity seemed to have reached steady-state levels by year 2, in dog 1 the decay from peak seemed to continue beyond year 2 and stabilized ∼4 years post gene transfer. The reasons for this additional decline are currently unknown. A significant loss of transduced cells due to inflammatory/immune reactions was ruled out, as we did not observe any clinical, biochemical, or diagnostic sign of inflammation of the CNS during the in-life portion of the study. Sacrifice of the animals 7 years after gene transfer gave us the opportunity to comprehensively analyze vector distribution and transgene expression and activity at tissue level. In general, dog 1 showed slightly lower levels of VGCN in the CNS compared to dogs 2 and 3. However, this difference was not reflected in significant differences in *Sgsh* mRNA expression levels, which were fairly similar across the encephalon of all 3 dogs. Although sulfamidase expression was detectable in most deep brain areas, the striatum, epithalamus, and thalamus showed very low to no detectable mRNA levels, which could be a limitation of intra-CSF-based gene therapy in transducing deep brain structures.^34^^,^^38^ It should be borne in mind that MPSIIIA is caused by mutations in a secretable lysosomal enzyme that can correct the enzymatic deficit of non-transduced cells through cross-correction. Preclinical and clinical data with intra-CSF ERT for several different secretable lysosomal enzymes^52^^,^^69^, ^70^, ^71^, ^72^, ^73^, ^74^, ^75^, ^76^ suggest that therapeutic efficacy can be expected not only from direct vector transduction but also from the presence of the enzyme in the CSF. Differences in transgene expression were somewhat more pronounced in the spinal cord and DRGs. For example, dog 3 seemed to have slightly higher levels of *Sgsh* mRNA expression in spinal cord, but it is difficult to interpret whether those differences are of significance, as CSF SGSH activity levels were very similar in dogs 2 and 3. In any case, the comparative analysis of vector distribution and expression data across the animals argued against transgene silencing as an explanation for the different behavior along the years of CSF SGSH in the different dogs.

The levels in CSF of a transgene expressed and secreted by transduced CNS cells is likely to result from a complex interaction of factors, including rate of production and clearance, both of which could be influenced by physiological and pathological processes. Along the years, a certain percentage of transduced cells could be lost not necessarily due to toxicity or inflammation but to physiological cell turnover. In this regard, after intra-CSF administration of AAV9 vectors encoding the reporter gene GFP we observed transduction of neurons and astrocytes but also of ependymal and leptomeningeal cells.^34^ In contrast to neurons, astrocytes as well as ependymal and leptomeningeal cells have a slow turnover.^77^, ^78^, ^79^, ^80^ In particular, the impact of the loss of ependymocytes and leptomeningeal cells that are in direct contact with the CSF as a monolayer lining the ventricles and meninges, respectively, would most likely be undetectable through the analysis of the expression of the transgene in tissue punches. This could explain why, despite the observed differences in SGSH levels in CSF, there are no significant differences in tissue *Sgsh* expression among the dogs, with >30 samples/dog of encephalon analyzed. Differences in expression and SGSH activity are more pronounced in the spinal cord, where dog 1 shows the lowest levels for both readouts; it could be the case that cells from the spinal cord are major contributors to the output of SGSH to the CSF. Alternatively, it may be the case that transduction was overall less efficient in dog 1. Because in general dog 1 shows lower VGCN, we consider it unlikely that vector silencing underlies the more pronounced drop in CSF SGSH in this dog. Other factors, such as changes in the volume of CSF of a magnitude that could impact the concentration of the enzyme in this fluid over the course of the life of dog 1, seem unlikely.^81^^,^^82^

The administration of the therapeutic product to the most challenging-to-treat anatomical compartment—the CNS—guarantees therapeutic levels of the transgene in the organ most severely affected. Additionally, the escape of vector to the circulation and subsequent transduction of the liver could provide a peripheral source of the therapeutic protein. Indeed, we were the first to report whole-body correction of a LSD upon delivery of AAV9 vectors to the CSF of MPSIIIA mice,^34^ and we have since extended our observations to 3 other indications.^35^^,^^39^^,^^45^ In principle, peripheral efficacy would depend on the maintenance of a certain level of hepatic transduction, sufficient to support the production of therapeutic levels of the transgene product. In the 3 dogs treated in this study, we found variable levels of transduction in the liver leading to different levels of transgene expression. Although vector genomes were undetectable in liver biopsies of dog 1, dog 2 and dog 3 presented vg/dg in all samples. Noticeably, the VGCN remained stable from the time of the biopsies (48 and 54 months) to the time of sacrifice (82 months) in all dogs. The correlation with sulfamidase activity measured in post mortem tissue extracts was less clear. Whereas in the CSF the baseline amount of SGSH activity is very low, which allows for reliable detection of increases that can be attributed to the administration of sulfamidase-encoding vectors, endogenous levels of SGSH activity in liver parenchyma, and CNS parenchyma, are high, which makes interpretation of results more challenging, as small variations can be masked by the high endogenous activity.

Given the absence of any signs that would suggest loss of vector genomes from the liver of dog 1, our observations might be reflecting true differences in liver transduction efficacy. Evidence supporting this hypothesis was provided by the longitudinal quantification of anti-AAV9 NAbs in paired CSF and serum samples for all dogs. Although the 3 dogs were negative for NAbs in the CSF at baseline, which explained the similar level of transduction efficacy in the CNS, dog 1 showed the presence of NAbs in circulation at 1:5–1:10 titer. Although low, this titer has been described to be sufficient to block liver transduction in large animals and humans.^83^^,^^84^ The absence of NAbs in the CSF of an animal seropositive for AAV9 is not surprising, given the asymmetrical distribution of NAbs across the BBB, even in patients in whom mild disruption of the BBB is expected.^34^ It is actually possible that dog 1 did have some NAbs in the CSF prior to vector administration but the levels fell below the detection limit of our NAb assay. It is also possible that, even if undetectable, those low levels of NAbs in CSF partially affected transduction in dog 1, which seemed to show more regions of the CNS negative for AAV vector genomes than the other dogs. Evidently, if present, the greatest proportion of those NAbs were neutralized by the high dose of vectors delivered directly to the CSF. The efficient transduction of the CNS in the presence of peripheral NAbs observed in dog 1 is in agreement with our earlier work; we showed that in dogs pre-immunized by systemic exposure to AAV it is possible to achieve successful CNS transduction with minimal loss of efficacy, albeit peripheral transduction is completely blocked.^34^^,^^39^

If efficacy in mild peripheral MPSIIIA disease was not achieved because of the lack of transduction of the liver, there would always be the possibility of providing patients with intravenous ERT, at expectedly lower doses than those required to treat severe CNS pathology, and most likely under a treatment regime more favorable to patients. Finally, it should be noted that, as the CSF ultimately drains into the circulatory and lymphatic systems, a certain amount of the therapeutic protein could also pass to the periphery. In this regard, studies conducted in a dog model of late infantile neuronal ceroid lipofuscinosis (Batten disease) found significant amounts of the soluble lysosomal enzyme tripeptidyl peptidase 1 (TPP1) in heart and spleen after delivery of TPP-encoding AAV2 vectors to the CSF, an AAV serotype with minimal peripheral dissemination.^59^

Besides the documentation of steady levels of therapeutic transgene through multiple years, another important outcome of this study is the demonstration of the good tolerability and long-term safety of the approach. Animals remained in an excellent health status throughout the study. We did not document any clinically relevant increase in protein levels or WBC counts at any of the time points at which CSF was sampled. Likewise, no clinically relevant alterations in biochemical or hematological parameters could be attributed to the procedure or test article administration. As further proof of long-term safety, in-life MRI and ultrasound imaging, as well as histopathological studies of post mortem specimens, revealed no evident abnormalities in the architecture of the encephalon, spinal cord, DRGs, or liver. DRG toxicity, characterized by mononuclear cell infiltrate, gliosis, neuronal loss, and axonopathy, has recently been identified as a common, albeit low in magnitude, histological finding following i.v. and intra-CSF but not intraparenchymal administration of high doses of AAV vectors to NHPs.^47^, ^48^, ^49^, ^50^ Toxicity is dose dependent^48^ and can be attenuated by use of microRNA sequences that de-target expression from DRGs,^85^ and frequency is higher after intra-CSF delivery, likely because of the use of tissue-specific promoters when targeting peripheral organs.^48^ Although not completely elucidated, all evidence seems to point toward high levels of transgene expression due to highly efficient transduction of ganglia sensory neurons as the root cause of toxicity (ganglia have fenestrated capillary, and sensory neurons are exposed to CSF).^34^ Indeed, the VGCN in the DRG was lower compared to other short-term studies conducted by us and others after intra-CSF delivery of AAV9 to NHPs.^34^^,^^50^^,^^86^ The observation of higher VGCN shortly after vector delivery is a constant observation in the field of *in vivo* gene transfer with AAVs, irrespective of the transgene and target organ. Whether the difference in VGCN reported in short- versus long-term studies reflects non-functional vector genomes that were not taken up by cells and are cleared with time, stabilization of only an initial fraction of the vector genomes in the nucleus, or turnover of some of the targeted cells remains to be determined for each organ.^5^^,^^68^^,^^87^ Importantly, no clinical findings have been reported to date from clinical trials, and the findings in NHP studies remain a histological finding, and no clinical signs have been correlated after analysis of 256 animals.^48^ When we analyzed the DRGs of our dogs, we could not find any histological signs of DRG toxicity. The reasons for this may be several: (1) Findings have been reported mostly in NHPs,^47^, ^48^, ^49^, ^50^ with only one report in a different species (neonatal piglets^49^). To the best of our knowledge, this is the first report investigating AAV-mediated DRG toxicity in dogs after such a long follow-up of 7 years. A recent study reported similar findings at 2.5 years of follow-up after intra-CSF AAV9 treatment in Krabbe disease dogs^88^; hence, it is possible that dogs’ DRG neurons are not as highly transduced as primates’. (2) A dose-effect relationship has been clearly established, and the doses used in the current study are lower than those used in the smaller cynos and rhesus monkeys.^48^ (3) The timing of our analysis is posterior to the development and resolution of toxicity. In any case, the close clinical follow-up of the dogs in this study, which were subjected to extensive neurological evaluations, suggests that, if present, DRG toxicity had no clinical consequences.

In the present study, we chose to use healthy beagle dogs as a large animal model. Although there is a spontaneous dog model of MPSIIIA for which an experimental colony has been established (MPSIIIA New Zealand Huntaway dog), its use does not provide any additional benefits for the long-term evaluation of sulfamidase expression and safety. On one hand, the clinical manifestations and evolution of the disease seem to differ substantially between humans and dogs, which mostly present signs of cerebellar disease, with minimal or no behavioral alterations or somatic disease.^89^, ^90^, ^91^ On the other hand, the New Zealand Huntaway dog bears a frameshift mutation with premature codon stop^92^ resulting in a complete lack of sulfamidase expression, which renders this animal immunologically intolerant to the sulfamidase protein. Acute reactions to the human protein have been well documented in ERT studies performed in this animal model.^61^^,^^93^ The intolerance to the canine sulfamidase protein, even if expressed from an AAV vector, can be predicted from observations made in earlier studies in hemophilia B dogs bearing null mutations and receiving AAVs encoding for canine factor IX (FIX).^94^ To enable long-term evaluation of the treatment in New Zealand Huntaway dogs, the co-administration of immunosuppression would be necessary, which could not only compromise the life of dogs but also introduce a confounding variable into the evaluation of possible adverse effects. Moreover, >90% of the mutations in the human *SGSH* gene described so far are missense mutations, and the report on the largest cohort of MPSIIIA patients studied up to now showed that >98% of subjects were carriers of at least one allele corresponding to a missense mutation.^7^ Thus, most patients are expected to have some degree of tolerance to sulfamidase, and therefore healthy beagle dogs are a better predictor of possible outcomes than the New Zealand Huntaway dog.

In conclusion, our study provides strong evidence of the feasibility and long-term durability and safety of using AAV9 vectors to deliver genes to the CNS. After the administration of clinically relevant doses of vector, the detection of sustained, significant levels of sulfamidase in the CSF and of widespread transgene expression in the CNS, PNS, and liver of treated dogs in the absence of any signs of toxicity after many years of follow-up provides strong evidence supporting the use of intra-CSF AAV9-mediated gene therapy to treat MPSIIIA and other neurodegenerative disorders.

## Materials and methods

### Animals

Male healthy beagle dogs (n = 3; dogs 1 and 2: 9 months, dog 3: 8 months of age) were purchased from Isoquimen (Spain) and housed at the Veterinary School of the Universitat Autònoma de Barcelona (UAB). Animals’ health was monitored through clinical, hematological, and biochemical examination. Animals were fed once daily at 9:00 AM with 30 g/kg body weight standard dry food (Elite Nutrition, Nestle). For general anesthesia, dogs were premedicated with an intramuscular administration of 0.05 mg/kg of acepromazine (Equipromacina, Fatro Ibérica) and 0.2 mg/kg of butorphanol (Torbugesic, Zoetis). Thirty minutes later, induction was performed by i.v. administration of 4 mg/kg of propofol (PropoVet, B. Braun Medical) and 0.5 mg/kg of diazepam (Valium). After endotracheal intubation, anesthesia was maintained by inhalation of 2% isoflurane (IsoVet, B. Braun Medical) in 100% oxygen. Throughout all surgical procedures, temperature, cardiac and respiratory frequency, capnography, arterial pressure, pulse, and electrocardiography were monitored with a multifunctional patient Vet Care monitor (B. Braun Medical). The neurological evaluation of injected dogs was performed by a diplomate veterinary neurologist from the Veterinary Clinical Hospital of the UAB. All experimental procedures were approved by the Ethics Committee for Animal and Human Experimentation of the UAB.

### AAV vector production

AAV expression cassette was generated by cloning the cDNA of optimized canine sulfamidase (GeneArt, Life Technologies) into an AAV backbone plasmid containing the ubiquitous CAG promoter (hybrid of cytomegalovirus [CMV] enhancer and chicken β-actin promoter) and the rabbit beta-globin polyA signal (rβGpA). AAV9 vectors were produced by triple transfection of HEK293 cells and purified with an optimized purification protocol with double cesium chloride gradient ultracentrifugation that results in vector preps of high purity with negligible amounts of empty capsids.^95^ Vectors were titered by quantitative real-time PCR and stored at −80°C until use.

### CSF collection and intracisternal administration of vectors

CSF collection and delivery of vectors was performed as previously described.^34^ Briefly, anesthetized dogs were positioned in lateral recumbency, and a 22-gauge needle was introduced into the cisterna magna between the occipital bone and the C1 vertebra. Right placement of the needle was confirmed by spontaneous flow of crystal-clear CSF, which was collected in sterile tubes and stored at −80°C until further analysis. For vector delivery, a syringe connected to a fluid extension catheter tube with a 3-way stopcock was attached to the needle and 1 mL total volume of vector solution was injected over a period of 15 s. The dose used (2 × 10^13^ vg/dog) was obtained by scaling up the dose previously found to be effective in mice,^34^ based on body weight of mice compared to dogs.

### Metabolite analysis and cell counts

General laboratory parameters and CSF TP content were measured by spectrophotometry with a Cobas Mira Analyzer (Roche) at the Clinical Biochemistry Lab of the Veterinary Clinical Hospital, UAB. Hemograms were analyzed with a SCIL VetABC hematology analyzer and CSF cell counts, WBC and RBC, were manually counted with a Neubauer chamber by the Clinical Hematology Lab.

### Ultrasonography and magnetic resonance imaging

For ultrasound imaging, anesthetized dogs were positioned in dorsal recumbency and an abdominal ultrasound was performed with a high-frequency linear transducer (LA523, linear array, 18 MHz, MyLab70, Esaote). For brain MRI, anesthetized dogs were positioned in ventral recumbency and scans were performed with a 0.2-T permanent open magnet system (Vet MR, Esaote). Technical parameters were (1) T1-weighted images in transverse and sagittal planes: TR = 800 ms, TE = 26 ms, FOV = 180 × 180 mm, thickness = 4.0 mm, spacing = 0.4 mm, matrix = 224 × 224, and NEX = 3; (2) T2-weighted images in transverse, sagittal, and dorsal planes: TR = 3,000 ms, TE = 80 ms, FOV = 180 × 180 mm, thickness = 4.0 mm, spacing = 0.4 mm, matrix = 224 × 224, and NEX = 1; and (3) fluid-attenuated inversion recovery (FLAIR) images in transverse planes: TR = 6,960 ms, TE = 80 ms, TI = 1,800 ms, FOV = 200 × 200 mm, thickness = 4.0 mm, spacing = 0.4 mm, matrix = 192 × 192, and NEX = 1. In addition, contrast-enhanced T1-weighted images of transverse and sagittal planes were acquired after intravenous administration of paramagnetic contrast medium (0.1 mmol/kg gadopentetate dimeglumine [Magnevist, Bayer]). For spinal cord MRI, anesthetized dogs were positioned in dorsal recumbency and scans were performed with a 0.4-T scanner (Aperto Lucent, Hitachi Medical). Technical parameters were (1) T1-weighted images in transverse and sagittal planes: TR = 653 ms, TE = 23 ms, FOV = 250 × 250 mm, thickness = 4.0 mm, spacing = 0.5 mm, matrix = 288 × 220, and NEX = 2 and (2) T2-weighted images in transverse, sagittal, and dorsal planes: TR = 3,100 ms, TE = 80 ms, FOV = 150 × 150 mm, thickness = 4.0 mm, spacing = 0.5 mm, matrix = 256 × 224, and NEX = 6. Contrast-enhanced T1-weighted transverse and sagittal images were also obtained.

### Sample collection

Forty-eight and fifty-four months after vector administration, percutaneous ultrasound-guided liver biopsies were obtained under general anesthesia. Briefly, abdominal hair was clipped, and a microconvex sector transducer operating at 6 or 7 MHz was used to guide liver sampling with a 14 gauge × 9 cm Tru-Cut biopsy needle (J0528, SuperCore Biopsy Needle, Argon Medical Devices).

Eighty-two months after vector administration, dogs were pre-medicated by intramuscular injection of 0.2 mg/kg butorphanol (Torbugesic, Zoetis) and 0.05 mg/kg acepromazine (Equipromacina, Fatro Ibérica). Thirty minutes later, euthanasia was performed with an overdose of pentobarbital (150 mg/kg, Dolethal) administered i.v. The encephalon, the spinal cord, the DRGs, and multiple somatic tissues were collected and either formalin-fixed or snap-frozen and stored at −80°C until use.

### Sulfamidase activity

CNS and liver samples were sonicated in 100–500 μL of Milli-Q water. Enzymatic activity was assayed in supernatants of sonicated samples or CSF with a 2-step protocol using 4-methylumbelliferone-derived fluorogenic substrate (4-MU), as previously described.^96^ Briefly, 30 μg of TP or 10 μL of CSF was first incubated with 10 mmol/L 4-methylumbelliferyl-2-sulfamino-2-deoxy-alpha-d-glucopyranoside sodium salt (4-MU-αGlcNS, Enantia, Spain) and 10 μL of complete solution (Roche) for 17 h at 47°C. The second incubation was carried out after the addition of 6 μL of PiCi buffer (0.4 M Na_2_HPO_4_·12H_2_O-0.2 M tris-sodium citrate 2-hydrate, pH 6.7 + 0.025% [w/v] Triton X-100) in the presence of 10 U/mL of α-glucosidase (Sigma) in 0.2% BSA (pH and heat inactivated, Sigma) for 24 h at 37°C. After the second enzymatic reaction was stopped by increasing the pH, the released fluorescence was measured with a Synergy HTX fluorimeter (BioTek Instruments). Sulfamidase activity was normalized against CSF volume (expressed as nmol/17 h/mL) or TP content (expressed as nmol/17 h/mg protein) quantified by Bradford assay (Bio-Rad).

### Vector genome copy number

CNS, PNS ganglia, or peripheral tissue samples were digested overnight (ON) at 56°C in 300 μL of Tissue Lysis Solution supplemented with Proteinase K (0.2 mg/mL). Total DNA was isolated from supernatants with the MasterPure DNA Purification Kit (Lucigen). DNA was resuspended in distilled water and quantified with a NanoDrop ND-1000 spectrophotometer (NanoDrop). VGCN in 40 ng of total DNA was determined by quantitative real-time PCR using the LightCycler 480 Probes Master (Roche). For analysis of liver biopsies, primers and probe specific for sequence within the optimized canine sulfamidase cDNA were used: forward primer: 5′-GCA TCA TCG GCA AGA AAC AC-3′; reverse primer: 5′-CAG CTT GAT CCT GGT GAT GT-3′; probe: 5′-ACA CCG AAG AGA ACA GCA GCG T-3′. The analysis of terminal samples was done with primers and probe specific for the rβGpA sequence: forward primer: 5′- CTT GAG CAT CTG ACT TCT GGC TAA T-3′; reverse primer: 5′- GAT TTG CCC TCC CAT ATG TCC-3′; and probe: 5′-CCG AGT GAG AGA CAC AAA AAA TTC CAA CAC-3′. A reference standard curve was built from serial dilutions of a linearized plasmid bearing the CAG promoter, the optimized canine sulfamidase cDNA, and the rβGpA sequences spiked into 20 ng/μL of non-transduced dog genomic DNA.

### Sulfamidase expression

Total RNA was purified from tissues homogenized in TriPure Isolation Reagent (Roche) with the RNeasy Mini Kit (QIAGEN). RNA was quantified in a NanoDrop ND-1000 spectrophotometer (NanoDrop). cDNA was synthesized with a Transcriptor First Strand cDNA Synthesis Kit (Roche). quantitative real-time PCR was performed with the LightCycler 480 Probes Master (Roche) with the same optimized canine sulfamidase-specific primers and probe used for VGCN quantification in liver biopsies. The values obtained were normalized to the expression of the dog *RPLP0* gene: forward primer: 5′-ACC TCT TTC TTC CAG GCT TTA G-3′; reverse primer: 5′- CCA CTT TGT CTC CCG TCT TAA T-3′, probe: 5′- ACC ATT GAA ATC TTG AGT GAT GTG CAG-3′.

### Histology

Tissues were fixed for 12–24 h in 10% formalin, embedded in paraffin, and sectioned. In brain and spinal cord, H&E and Nissl staining and immunohistochemical staining against Iba1 and GFAP were performed in 5-μm sections. For DRG analysis, 3 serial 3-μm sections were obtained, at 3 different levels of the ganglia, with a separation between series of 50 μm. The first slide of each series was used for H&E staining, and the consecutive sections were used for immunohistochemical detection of Iba1 and GFAP, always following the same order of tissue sections. They were incubated overnight at 4°C with rabbit anti-GFAP (Z0334, Dako Cytomation) or goat anti-Iba1 (Ab5076, Abcam). The secondary antibodies were biotinylated goat anti-rabbit immunoglobulin G (IgG) (31820, Invitrogen) or biotinylated rabbit anti-goat IgG (E0466, Dako Cytomation). The ABC peroxidase kit (Pierce) was used for immunodetection, and sections were counterstained in Mayer’s hematoxylin. Images were obtained with an Eclipse E800 optical microscope (Nikon).

### Neutralizing antibodies

Anti-AAV9 NAb titers were determined in dog CSF and serum samples as previously described,^97^ with an *in vitro* neutralization assay, at ISGLOBAL, Barcelona Centre for International Health Research (CRESIB), Hospital Clínic, Barcelona.

### Statistical analysis

Results are expressed as mean ± SEM. Statistical comparisons for CSF sulfamidase activity were made with unpaired two-tailed t test. Statistical significance was considered if p < 0.05. Data on sulfamidase activity in tissues were represented with a confidence interval (CI) of 95% and 99% between control (non-injected dogs) and injected dogs.

## Supplemental information

Document 1. Figures S1–S9 and Tables S1–S5

Document 2. Article plus supplemental information
