# Supplementary material for: Seven-year follow-up of durability and safety of AAV CNS gene therapy for a lysosomal storage disorder in a large animal
Source: Mol Ther Methods Clin Dev. 2021 Oct 5;23:370–89. doi: 10.1016/j.omtm.2021.09.017 (PMC8550992; doi:10.1016/j.omtm.2021.09.017)
Supplement: Document 1. Figures S1–S9 and Tables S1–S5 [file mmc1.pdf]

OMTM, Volume *23*

Supplemental information

Seven-year follow-up of durability and safety of AAV CNS gene therapy for a lysosomal storage disorder in a large animal

Sara Marcó, Virginia Haurigot, Maria Luisa Jaén, Albert Ribera, Víctor Sánchez, Maria Molas, Miguel Garcia, Xavier León, Carles Roca, Xavier Sánchez, Joan Bertolin, Jennifer Pérez, Gemma Elias, Marc Navarro, Ana Carretero, Martí Pumarola, Anna Andaluz, Yvonne Espada, Sonia Añor, and Fatima Bosch

Figure S1

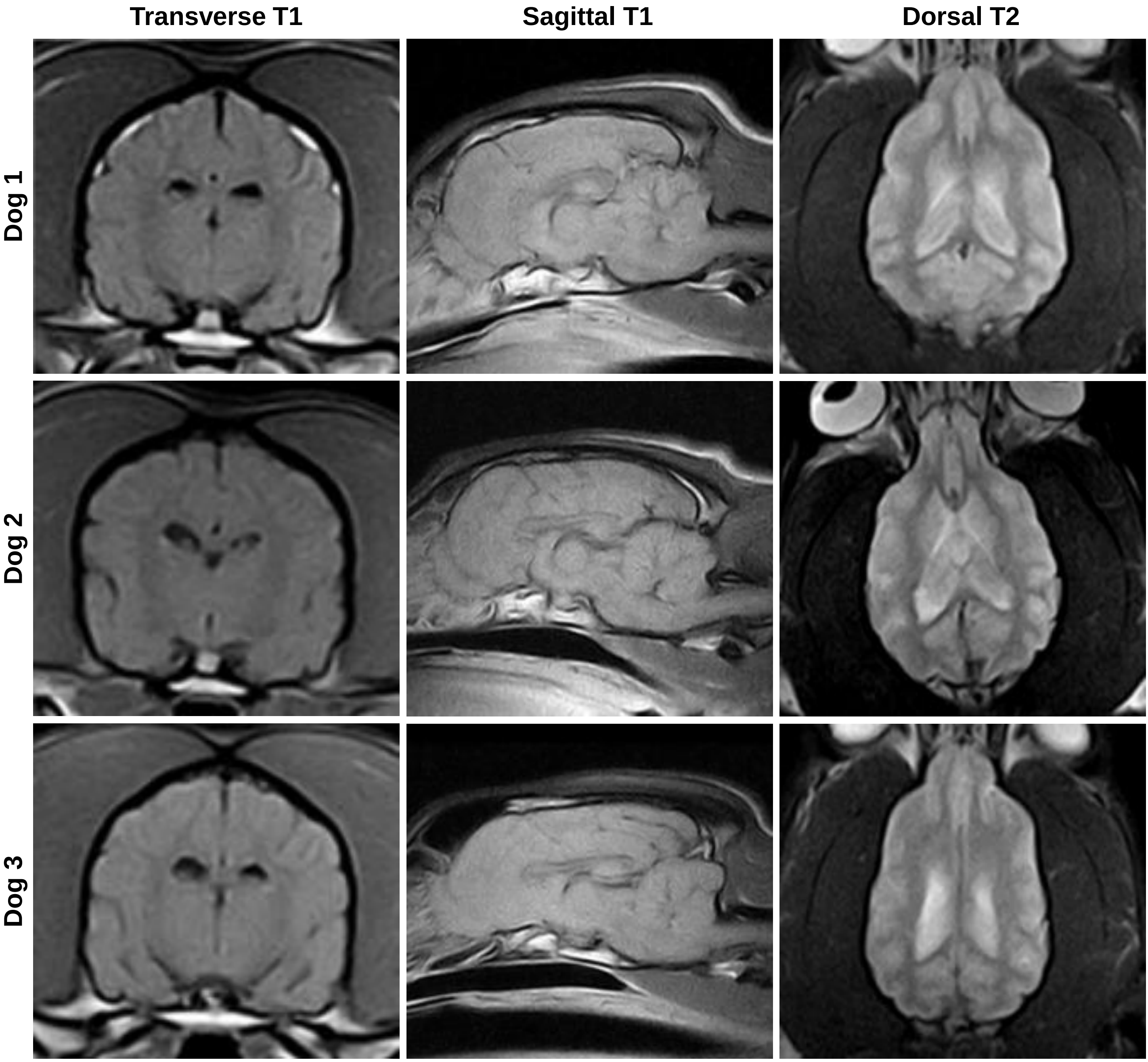

Figure S1. MRI imaging of the encephalon following intra-CSF AAV9-Sgsh delivery. MRI analysis of the encephalon of Dogs 1-3 performed 34 months after vector delivery. Scans were performed with a 0.2T permanent open magnet system. Left and middle panels correspond to transverse and sagittal T1-weighted images, respectively, obtained after intravenous administration of a gadolinium-containing contrast agent. Right panels correspond to dorsal T2-weighted images obtained prior to the injection of the contrast. No abnormalities were observed in the encephalon of any of the AAV9-Sgsh-injected dogs in any of the MRI sequences.

Figure S2

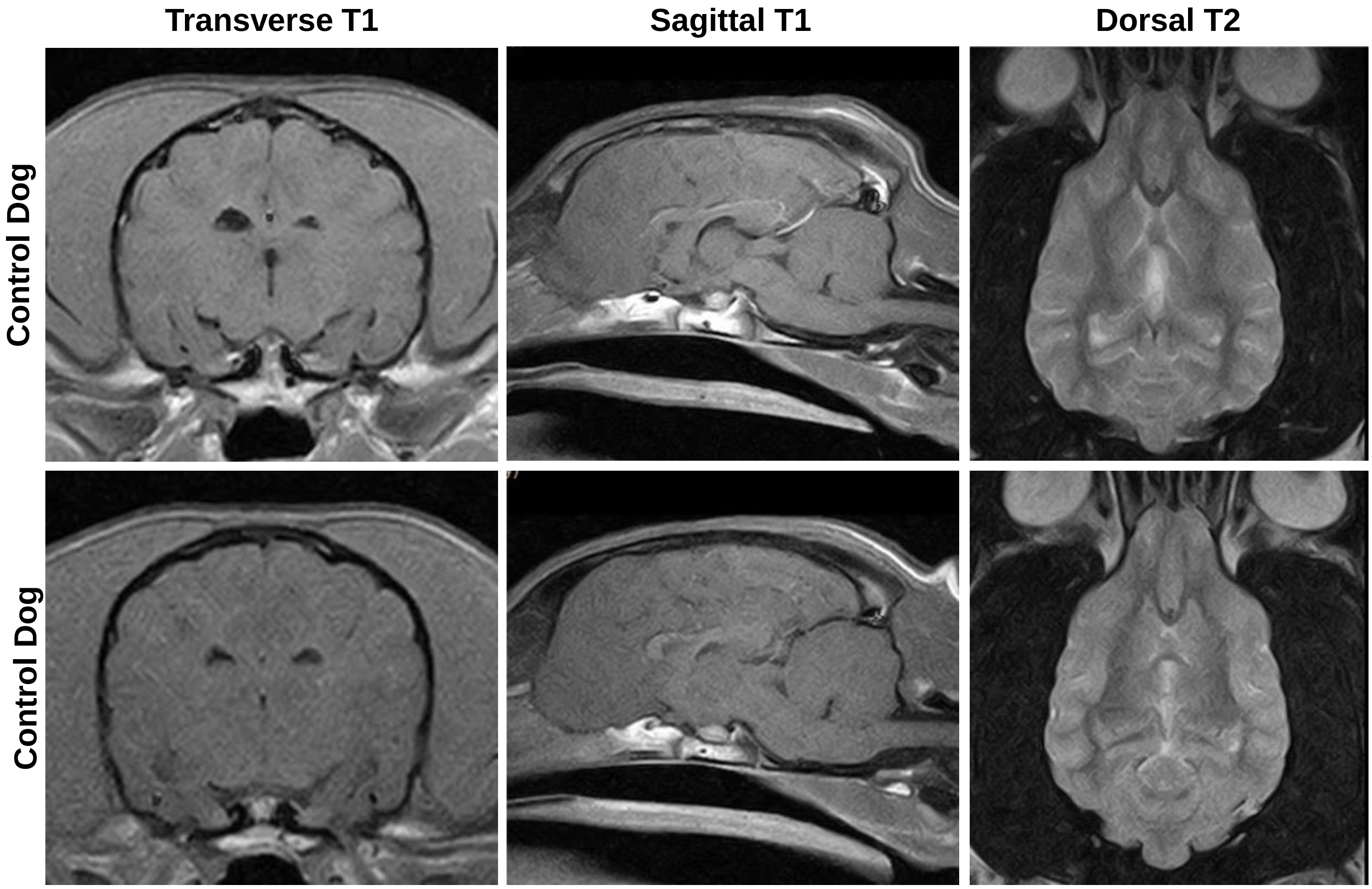

Figure S2. Control MRI images of the encephalon of uninjected healthy Beagle dogs. Representative images of the MRI analysis of the encephalon of two uninjected dogs, used as a reference images for the MRI analysis performed in AAV9-Sgsh-injected dogs. Scans were performed with a 0.2T permanent open magnet system. Left and middle panels correspond to transverse and sagittal T1-weighted images, respectively, obtained after intravenous administration of a gadolinium-containing contrast agent. Right panels correspond to dorsal T2-weighted images obtained prior to the injection of the contrast.

Figure S3

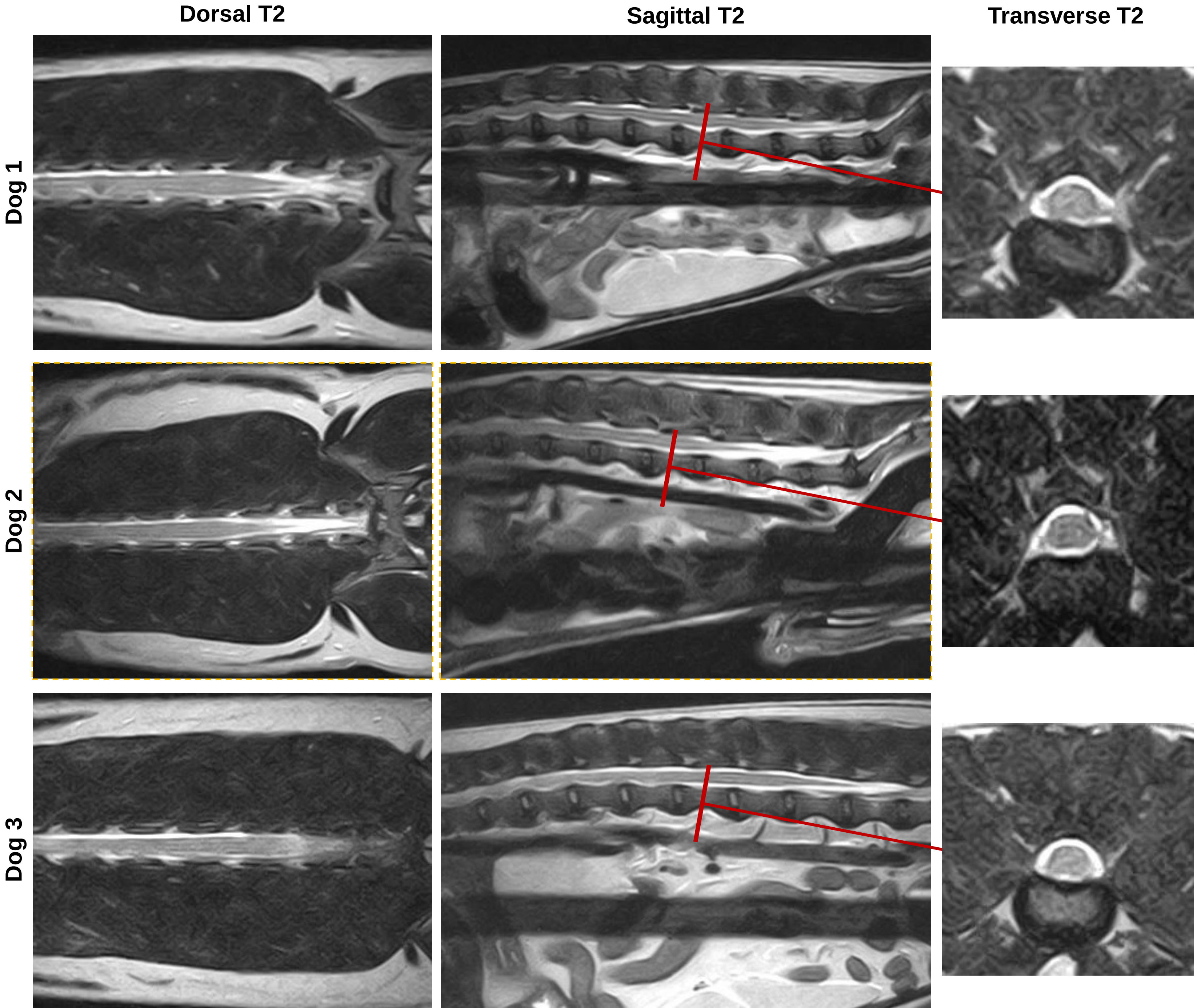

Figure S3. MRI of the spinal cord following intra-CSF AAV9-Sgsh delivery. MRI analysis of the spinal cord, performed 60 months after vector delivery, focused on the lumbosacral intumescence and the cauda equina regions as our previous work had determined these were the portions of the spinal cord most efficiently transduced following intra-CSF administration of AAV9 vectors to dogs34. A 0.4T scanner was used to obtain dorsal (left panels), sagittal (middle panels) and L2-L3 transverse T2-weighted images. Similar to the encephalon, no abnormalities could be identified in any of the dogs after systematic analysis of all the images obtained.

Figure S4

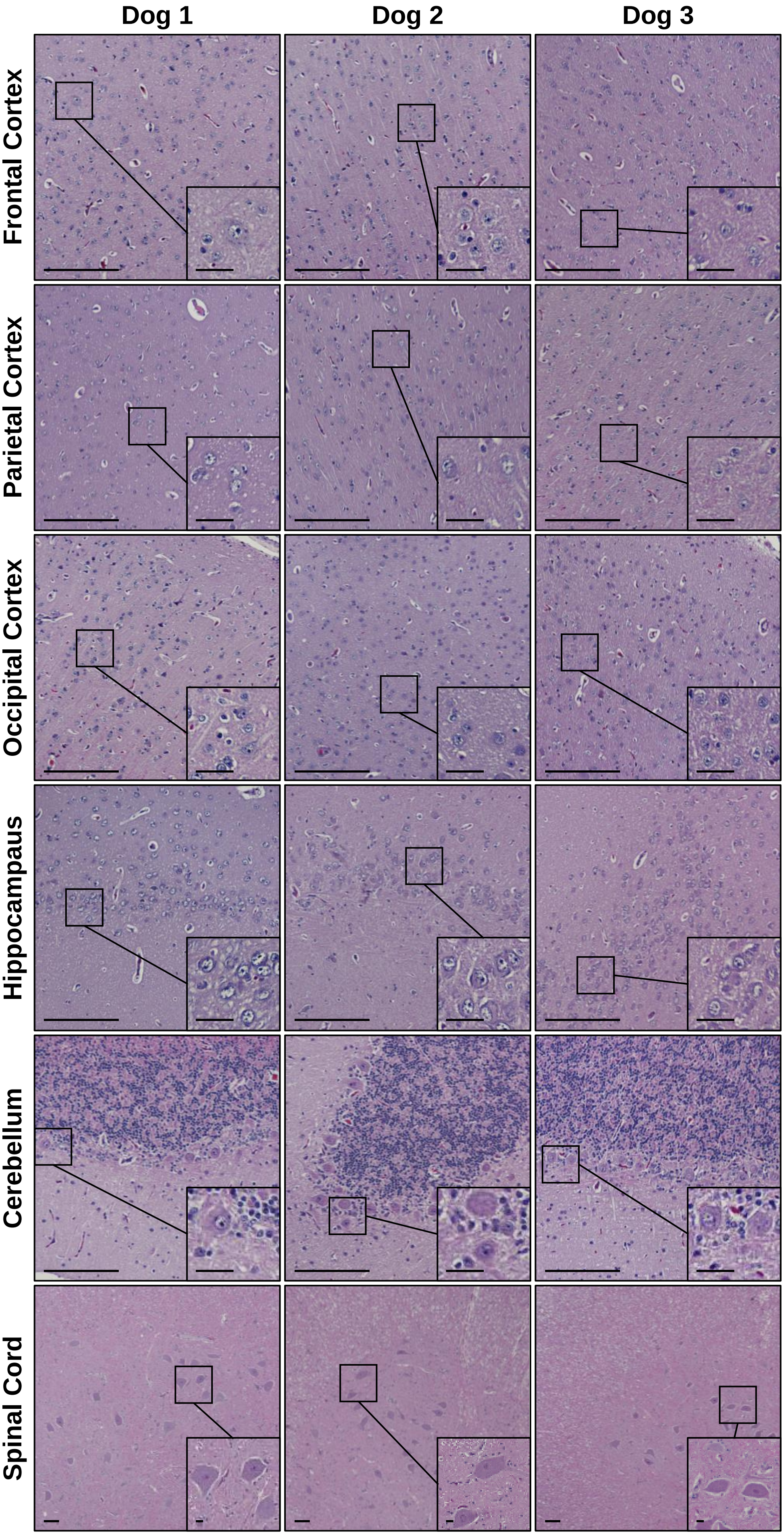
Dog 1
Dog 2
Dog 3
Frontal Cortex
Parietal Cortex
Occipital Cortex
Hippocampaus
Cerebellum
Spinal Cord

Figure S4. Histopathological study of the CNS following AAV9-Sgsh gene transfer. Representative images of the H&E staining of several areas of the encephalon and spinal cord of Dogs 1-3. Scale bars, 500 µm; insets, 100 µm.

Figure S5

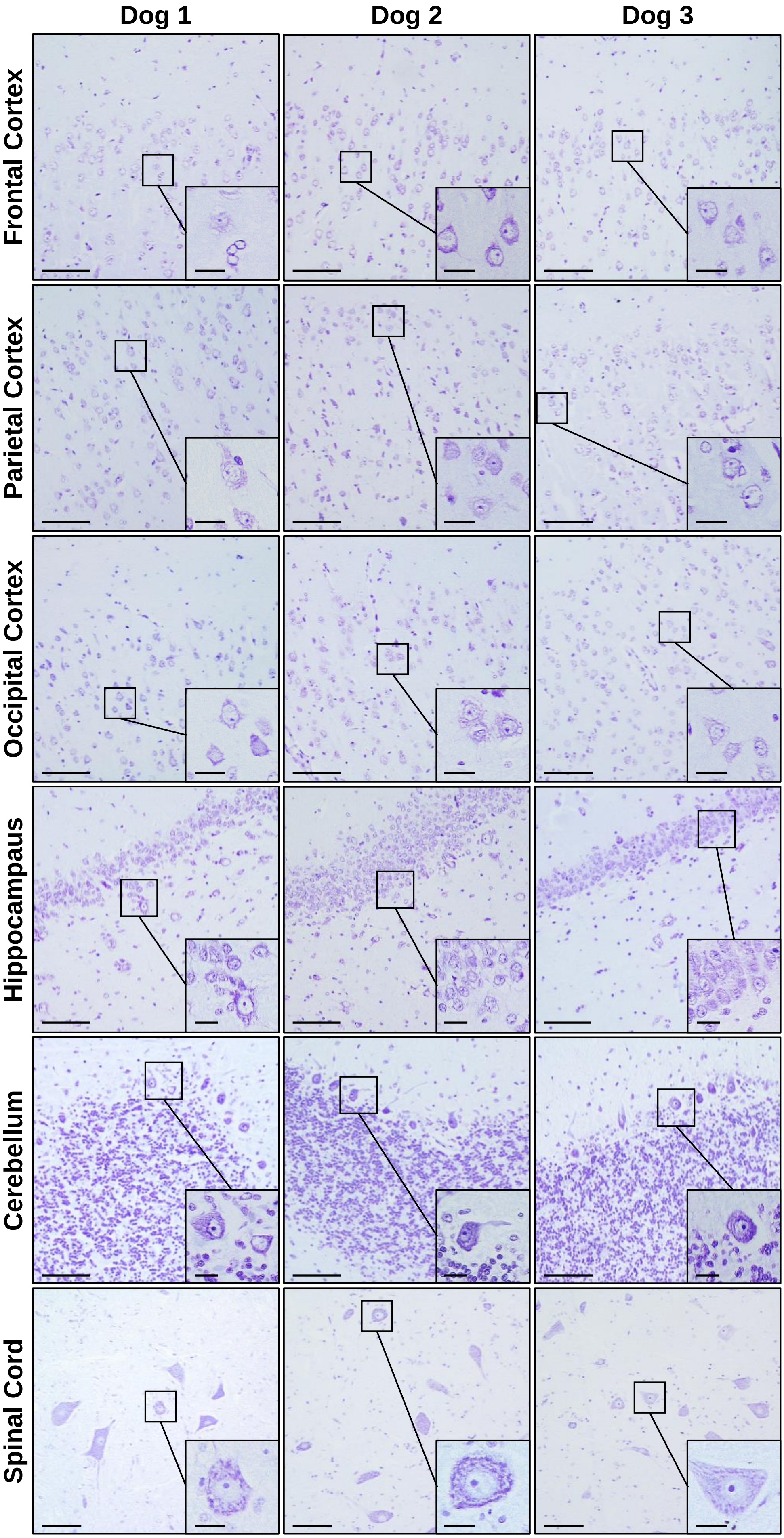
Dog 1
Dog 2
Dog 3
Frontal Cortex
Parietal Cortex
Occipital Cortex
Hippocampaus
Cerebellum
Spinal Cord

Figure S5. Histopathological study of the CNS after intra-CSF AAV9-Sgsh administration. Representative images of the Nissl staining of several areas of the encephalon and spinal cord of Dogs 1-3. Scale bars, 100 μm; insets, 20 μm.

Figure S6

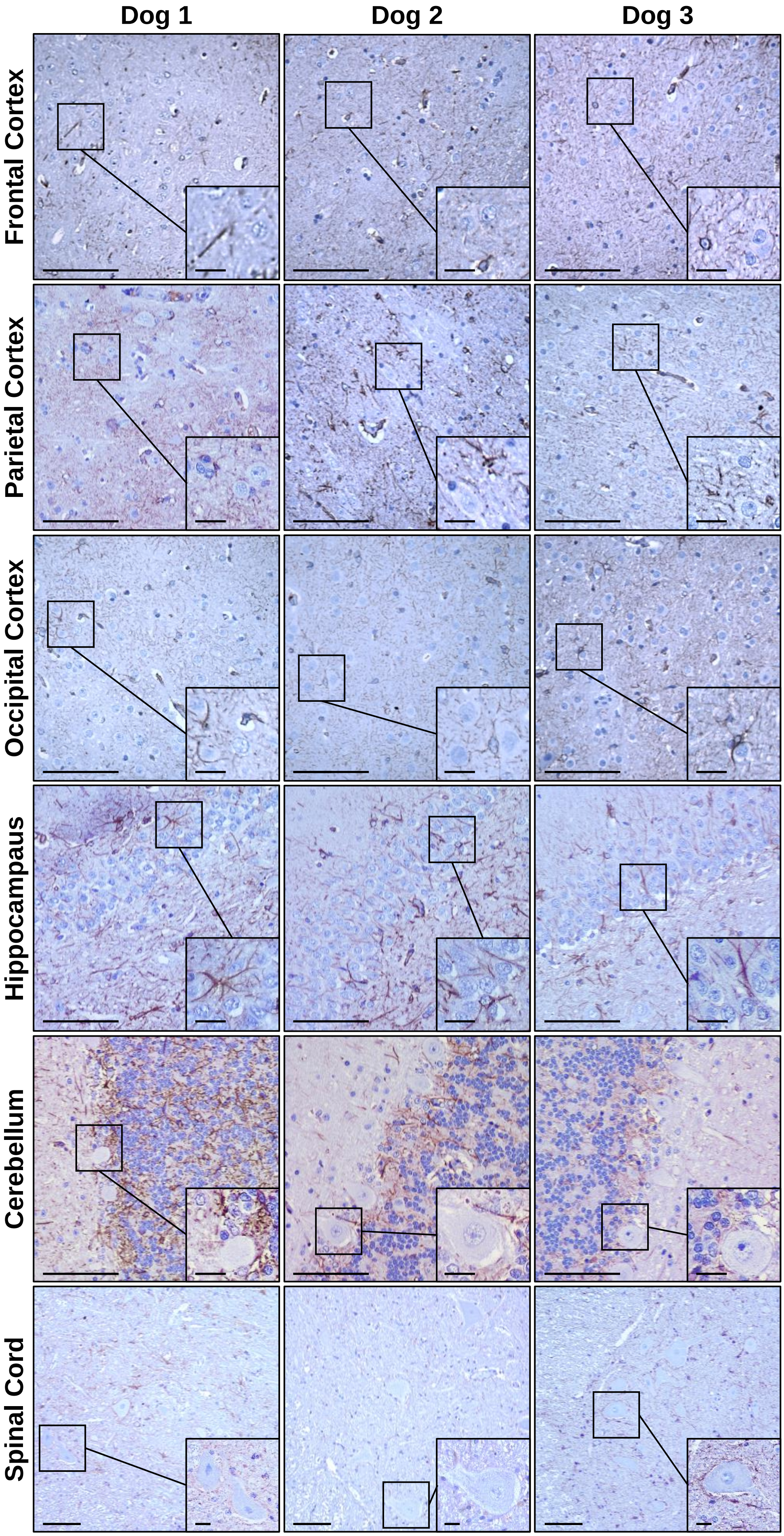
Dog 1
Dog 2
Dog 3
Frontal Cortex
Parietal Cortex
Occipital Cortex
Hippocampaus
Cerebellum
Spinal Cord

Figure S6. Assessment of adverse responses in the CNS after AAV9-Sgsh gene transfer. Representative images of the immunostaining with an antibody specific for the astrocyte marker GFAP performed on several areas of the encephalon and spinal cord of Dogs 1-3 seven years after gene transfer. Scale bars, 100 μm; insets, 20 μm.

Figure S7

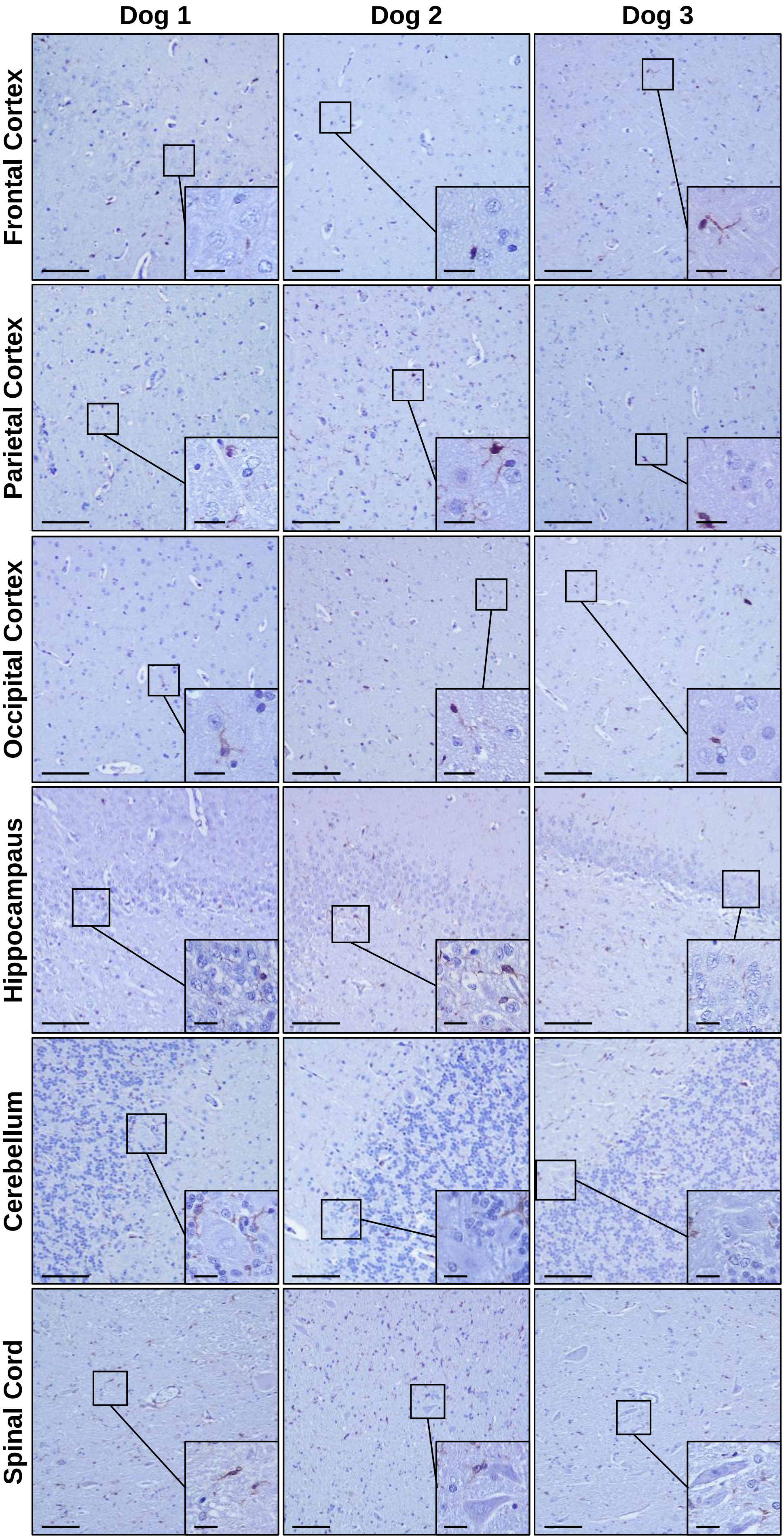
Dog 1
Dog 2
Dog 3
Frontal Cortex
Parietal Cortex
Occipital Cortex
Hippocampaus
Cerebellum
Spinal Cord

Figure S7. Assessment of adverse responses in the CNS following AAV9-Sgsh gene transfer. Representative images of the immunostaining with an antibody specific for the microglial marker Iba1 performed on several areas of the encephalon and spinal cord of Dogs 1-3 seven years after vector administration. 100 μm; insets, 20 μm.

Figure S8

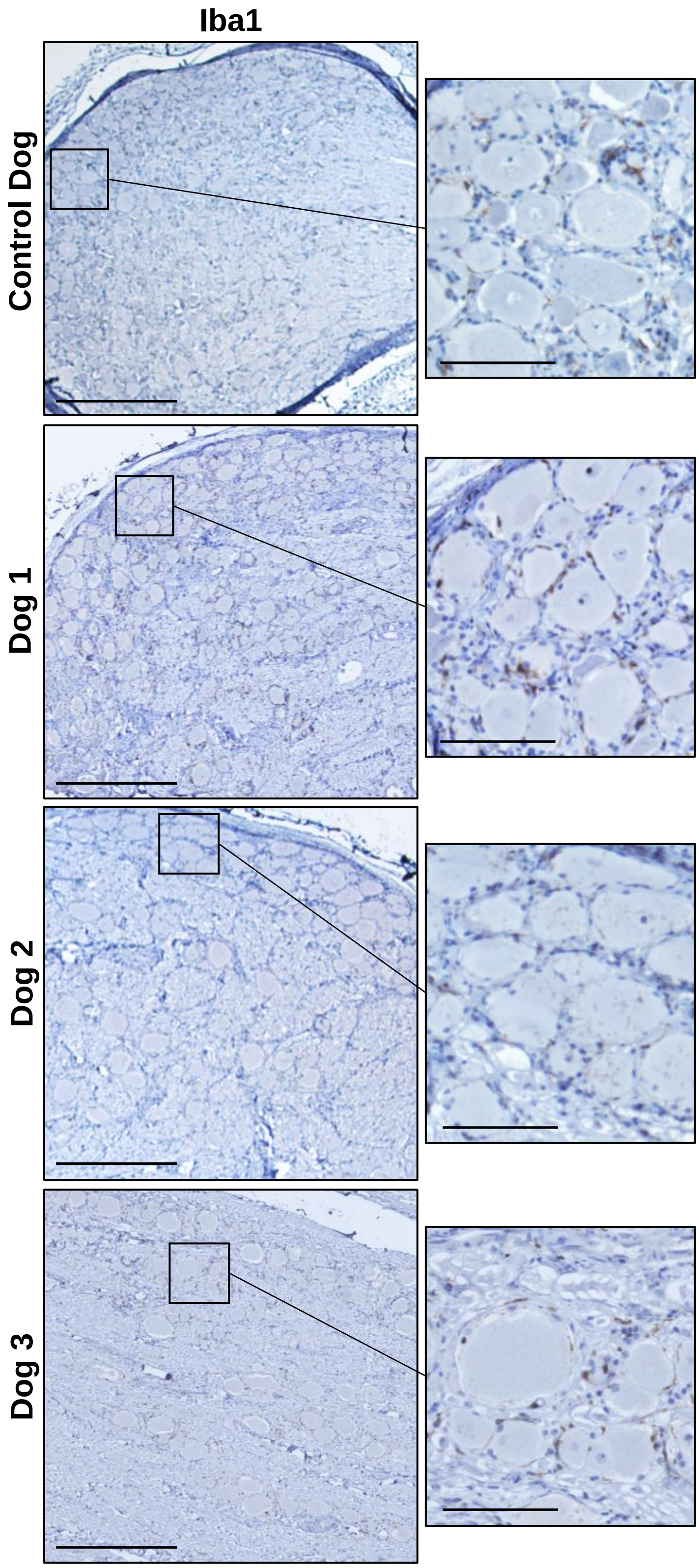

Figure S8. Histopathological analysis of dorsal root ganglia. Representative images corresponding to lumbar DRG obtained from Dog 1-3 and from a control, uninjected dog after immunohistochemistry for the microglial marker Iba1. Scale bars, 500 µm; insets, 100 µm.

Figure S9

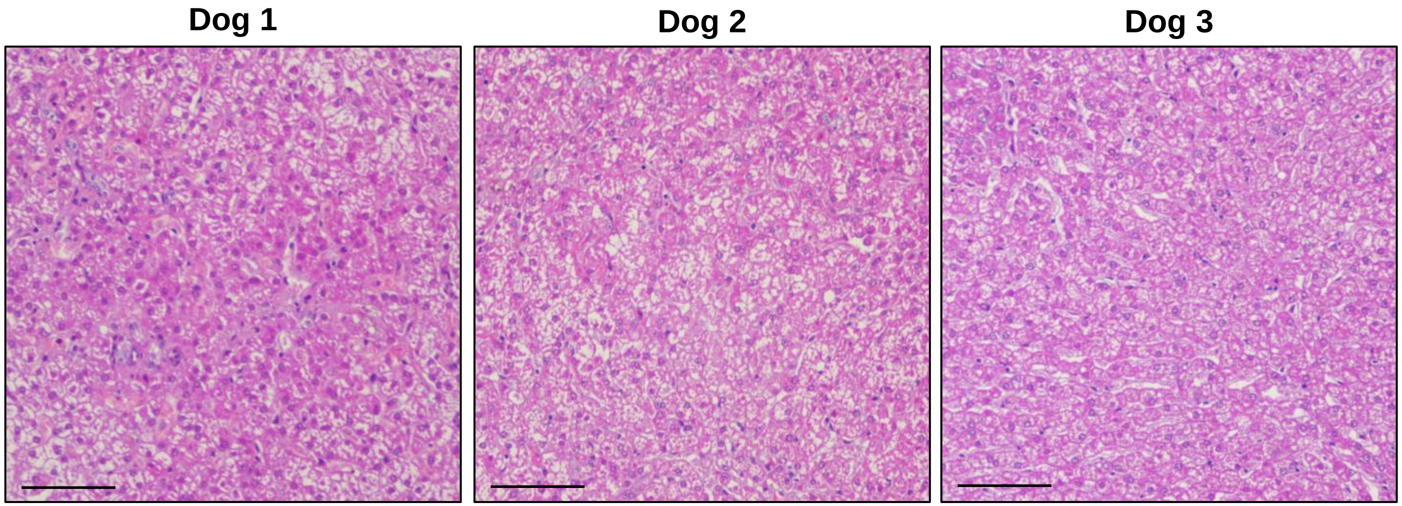

Figure S9. Histopathological study of the liver following AAV9-*Sgsh* gene transfer. Representative images of H&E staining of the liver of Dogs 1-3. Scale bars, 100 μm.

Table S1. Follow-up of clinical chemistry parameters measured in Beagle dogs that received a single intra-CSF injection of AAV9-*Sgsh*.

	Dog ID																				
	Dog 1							Dog 2							Dog 3						
**Parameters (reference values)**	**D0**	**D7**	**M1**	**M24**	**M48**	**M61**	**M82**	**D0**	**D7**	**M1**	**M24**	**M48**	**M61**	**M82**	**D0**	**D7**	**M1**	**M24**	**M48**	**M61**	**M82**
**ALT** (21 – 102 U/l)	36	31	54	39	41.6	32.6	36.3	48	53	54	52	60.7	47	79.2	26	25	29	43	43.2	49.2	81.5
**ALP** (20 – 156 U/l)	49.6	41.5	67.4	45.04	31.85	33.74	72.9	32.84	33.3	39.95	23.53	26.84	22.53	36.2	58.17	47.4	53.09	27.06	*19.85*	21.08	34.8
**GGT** (1.2 – 6.4 U/l)	*1*	2	2	NA	2	2	3.4	*1*	*1*	2	2	*1*	*1*	2.1	*1*	*1*	2	2	*1*	2	2.9
**Total Proteins** (5.4 – 7.1 mg/dl)	5.47	5.76	5.93	5.62	5.68	*5.11*	5.56	*5.36*	5.78	5.5	*5.22*	5.46	*4.85*	5.77	*5.36*	*5.34*	*5.06*	5.4	6.08	5.67	5.95
**Albumin** (2.6 – 3.3 g/dl)	2.87	2.87	2.94	2.74	3.08	2.82	2.79	2.89	3.04	2.92	*2.56*	2.67	2.63	2.61	3.17	2.99	2.84	3.15	*3.53*	*3.33*	3.28
**Gamma Globulin** (0.3 – 0.8 g/dl)	0.35	0.39	0.39	0.47	0.60	0.54	0.57	0.34	0.37	0.36	0.48	0.51	0.37	0.36	*0.20*	*0.22*	*0.21*	0.34	0.57	0.56	0.62
**A/G** (0.8 – 2)	1.10	0.99	0.99	0.95	1.18	1.23	1.01	1.17	1.11	1.13	0.96	0.96	1.18	0.82	1.44	1.27	1.28	1.40	1.39	1.42	1.23
**Total Bilirubin** (0.1 – 0.5 mg/dl)	0.13	0.15	0.16	*0.04*	0.14	0.14	0.1	0.17	0.17	0.19	0.14	0.14	*0.08*	0.14	0.18	0.11	0.18	0.29	0.13	0.16	0.11
**Glucose** (65 – 118 mg/dl)	*116.4*	*127.1*	*134.9*	*132*	94.9	85.8	103.8	*119.3*	*121.8*	*147.4*	112.3	96.7	*125.6*	96.6	115.2	*137.4*	112.2	100.4	98.2	102.8	102.7
**Total Cholesterol** (135 – 270 mg/dl)	166.9	168.5	196.3	170.8	178.1	192.7	251.1	162.8	175.2	191.4	172.5	257	266	244	154.4	160.8	174.8	*128.5*	149.5	147	156.9
**Urea** (21.4 – 59.9 mg/dl)	53.7	43.8	*70.2*	*60.7*	*74.2*	58.5	48.65	*64.2*	52.6	58.8	47.2	*62.3*	*80.2*	NA	22.9	21.7	23.9	22	26.4	27.5	*20.25*
**Creatinine** (0.5 – 1.5 mg/dl)	1.08	1.14	1.11	1.01	0.94	1.03	0.91	1.04	1.23	1.16	1.25	1.4	*1.76*	NA	0.67	0.79	0.77	0.86	0.82	1.1	0.79
**Ca^{2+}** (9 – 11.3 mg/dl)	10.1	10.7	10.9	9.9	9.4	9.4	9.56	10.1	10.8	10.7	10.6	10.2	9.7	10.82	10.9	10.6	10.8	9.9	10.1	9.3	9.74
**K^+** (4.37 – 5.35 mg/dl)	*3.39*	*3.42*	*3.77*	*3.25*	*4.16*	4.78	*3.96*	*3.27*	*3.37*	*3.58*	*3.68*	*4.25*	*4.27*	5.93	*3.18*	*3.2*	*3.53*	*3.14*	*3.6*	4.76	*3.46*
**P** (2.6 – 6.2 mg/dl)	5.07	3.93	5.64	3.19	4.72	*7.3*	3.41	4.48	4.16	5.15	4.69	5.09	5.67	*9.74*	*6.54*	4.67	*7.62*	3.43	4.68	*8.67*	3.14
**Na^+** (141 – 152 mg/dl)	*138.6*	141.8	146.7	*139.7*	*139.9*	143.1	147.5	*137.3*	142.3	149	*140.5*	*140.7*	144.1	146.3	*138.2*	142.9	147.9	143.9	141.3	143.8	148.6
**Cl^-** (105-115 mg/dl)	113.3	109.2	*115.6*	107	109.2	107.9	112.5	112.5	108.9	*119.4*	109.1	109.8	113.1	*115.2*	108.8	111	114.5	109.7	108.7	111.1	112.3

NA, sample not available. ALT = Alanine aminotransferase; ALP = Alkaline Phosphatase; GGT = Gamma-Glutamyl Transferase; A/G = Albumin/Gamma Globulin; Ca^{2+} = Calcium; K^+ = Potassium; P = Phosphorus; Na^+ = Sodium; Cl^- = Chlorine.

Blood samples were obtained regularly through the follow-up period. Values corresponding to samples obtained at baseline (D0 = day 0), at 1 week (D7 = day 7) and at 1, 24, 48, 61 and 82 months (M1, M24, M48, M61 and M82) post-vector delivery are shown. The values outside the reference interval are italicized.

Table S2. Follow-up of hematological parameters measured in Beagle dogs that received a single intra-CSF delivery of SGSH-encoding AAV9 vectors.

	Dog ID																				
	Dog 1							Dog 2							Dog 3						
**Parameters (reference values)**	**D0**	**D7**	**M1**	**M24**	**M48**	**M61**	**M82**	**D0**	**D7**	**M1**	**M24**	**M48**	**M61**	**M82**	**D0**	**D7**	**M1**	**M24**	**M46**	**M61**	**M82**
**Erythrocyte count** *(5.5 – 8.5 x10^6/µl)*	*4.84*	*4.79*	5.56	5.55	5.94	*5.23*	*5.08*	*4.96*	*5.06*	5.62	*5.17*	5.84	*3.85*	*2.07*	*5.32*	5.52	5.61	5.8	6.88	*5.49*	*5.13*
**Hemoglobin** *(12 – 18 g/dl)*	*11.8*	*11.6*	13	13.3	14	12.3	12.2	12	12.8	13.2	13.2	14.5	*9.6*	*5.04*	12.6	13	12.8	14.2	16.6	13	12.2
**Hematocrit** *(37 – 55 %)*	*33*	*34*	38	38	38	*36*	*35*	*34*	37	37	37	42	*30*	*13*	*36*	38	37	38	47	38	*32*
**MCV** *(62 – 77 fl)*	71.54	70.6	71.9	73.21	69.1	70.2	69.7	72.7	72.5	73.9	76.2	73	75.4	75.8	69.9	69.1	70.1	73.7	69.5	70	68
**MCHC** *(33 – 37 g/dl)*	34.1	34.1	*32.3*	34.7	35.3	34.2	34.5	33.3	34.9	*32.3*	34.4	35.3	34.4	34.4	33.9	33.9	*32.5*	34.5	35.4	34.8	35
**MCH** *(21.5 – 26.5 pg)*	24.4	24.2	23.4	24	23.6	23.5	24	24.2	25.3	23.5	25.5	24.8	24.9	24.3	23.7	23.6	22.8	24.5	24.1	23.7	23.8
**Leucocyte count** *(6000 – 17000 x/µl)*	9050	9270	11080	6360	9860	*5000*	6140	11500	12380	15110	9060	13470	*5750*	7080	9140	*5970*	6960	*5880*	8870	*5280*	*4660*
**Lymphocytes** *(1000 – 4800 x/µl)*	2806	2688	1551	1908	3451	1000	*921*	3450	3962	2267	2718	3906	1610	1628	2559	2567	2158	2176	3016	1901	1258
**Monocytes** *(150 – 1350 x/µl)*	272	185	776	*0*	690	300	614	*115*	248	*1662*	906	808	288	425	183	239	209	235	444	*53*	326
**Band neutrophils** *(0 – 300 x/µl)*	91	0	0	0	0	0	0	0	0	0	0	0	0	0	0	0	0	0	0	0	0
**Segmented neutrophils** *(3000 – 11500 x/µl)*	5521	5377	8642	4388	4930	3500	4175	7935	7057	11030	4983	7947	3565	4814	6307	*2985*	4385	3410	5145	3274	*2936*
**Eosinophils** *(100 – 1500 x/µl)*	362	1020	111	*64*	789	200	430	*0*	1114	151	453	808	288	212	*91*	179	*0*	*59*	266	*53*	140
**Basophils** *(0 – 200 x/µl)*	0	0	0	0	0	0	0	0	0	0	0	0	0	0	0	0	0	0	0	0	0
**Platelet count** *(200 – 500 x10^3 µl)*	*562*	*557*	*524*	269	299	281	325	482	*531*	332	279	311	264	352	*728*	*768*	*763*	406	291	327	280
**Reticulocyte count** *(0 – 60000)*	19360	NA	NA	NA	24948	29811	32512	29760	NA	NA	NA	28032	12320	10350	21280	NA	NA	NA	37840	24156	19494

NA, sample not available
MCV = Mean Corpuscular Volume; MCHC = Mean Corpuscular Hemoglobin Concentration; MCH = Mean Corpuscular Hemoglobin.
Blood samples were obtained regularly through the follow-up period. Values corresponding to samples obtained at baseline (D0 = day 0), at 1 week (D7 = day 7) and at 1, 24, 48, 61 and 82 months (M1, M24, M48, M61 and M82) post-vector delivery are shown. The values outside the reference interval are italicized.

Table S3. List of the neurological evaluations performed 52 and 82 months after intra-CSF delivery of AAV9-*Sgsh* vectors to the CSF of healthy Beagle dogs. All cranial nerves and spinal reflexes were evaluated for both the left and right side. Postural reactions were evaluated for both thoracic and pelvic limbs (in each left and right side).

**Dog ID**
**A) General observation**
Mental status
Posture
Gait
**B) Postural reactions**
Proprioception
Hopping
Wheelbarrowing
Extensor postural thrust
Hemistanding/Hemiwalking
Placing
**C) Cranial nerves**
Vision
Menace response
Pupillary size
Pupillary light reflex right eye
Pupillary light reflex left eye
Oculocephalic movements
Strabismus
Temporal/masseter muscles
Palpebral reflex
Facial reflexes (trigeminal nerve/facial nerve evaluation)
Swallowing
Tongue
**D) Spinal reflexes**
Triceps reflex
Biceps reflex
Flexor Thoracic limbs
Patellar reflex
Gastrocnemius muscle reflex
Flexor Pelvic limbs
Perineal reflex
Cutaneous trunci reflex
**E) Sensory evaluation**
Areas of hyperesthesia

Table S4. Vector genome copy number (VGCN) and mRNA expression in liver biopsies after intra-CSF delivery of AAV9-*Sgsh* vectors to Beagle dogs.

Dog ID	Virus injected intra-CSF	Liver	Time post-injection	oc*Sgsh* mRNA expression (AU)	vg/dg
Neg control	Non-injected	LML	-	ND	0.006
		LLL	-	ND	ND
		RML	-	0.037	ND
Neg control	AAV9-GFP	LML	7 days	ND	ND
		LLL	7 days	0.047	0.008
Dog 1	AAV9-*Sgsh*	LML	48 months	ND	ND
			54 months	0.033	ND
		LLL	48 months	ND	0.010
			54 months	ND	ND
Dog 2	AAV9-*Sgsh*	LML	48 months	1.410	2.094
			54 months	1.266	1.655
		LLL	48 months	0.221	1.117
			54 months	0.632	1.008
Dog 3	AAV9-*Sgsh*	LML	48 months	ND	0.089
			54 months	0.029	0.166
		LLL	48 months	0.092	0.010
			54 months	0.134	0.264

Results shown are the average of two needle-biopsies in the liver performed 48 and 54 months after intra-CSF administration. Results are shown as the mean ± SEM.
One non-injected dog and another dog injected in the cisterna magna with AAV9-GFP vectors were used as negative controls of the qPCR reaction.
ND, non-detectable; LML, Left Medial Lobe; LLL, Left Lateral Lobe; RML, Right Medial Lobe; vg/dg, vector genome/diploid genome; oc*Sgsh, optimized canine sulfamidase; AU, Arbitrary Units.*

Table S5. Determination of neutralizing antibodies against AAV9 vectors after intra-CSF delivery of $2x10^{13}$ vg of an AAV9 carrying the canine sulfamidase gene to healthy Beagle dogs.

		Dog ID					
		Dog 1		Dog 2		Dog 3	
		CSF	Serum	CSF	Serum	CSF	Serum
Time post-injection	Day 0	-	1:5 - 1:10	-	-	-	-
	Day 28	1:40	1:5120	1:80	1:2560	1:5	1:1280
	Month 15	1:20	1:2560	1:20	1:640	1:20	1:640
	Month 46	1:10	1:1280	1:10	1:320	1:20	1:640
	Month 82	1:10	1:1280	1:10	1:320	1:5	1:320
